# Supplementary material for: Extensive rewiring of the EGFR network in colorectal cancer cells expressing transforming levels of KRASG13D
Source: Nat Commun. 2020 Jan 24;11:499. doi: 10.1038/s41467-019-14224-9 (PMC6981206; doi:10.1038/s41467-019-14224-9)
Supplement: Supplementary file 1 — Supplementary Information [file 41467_2019_14224_MOESM1_ESM.pdf]

Supplementary Information

Supplementary Figures

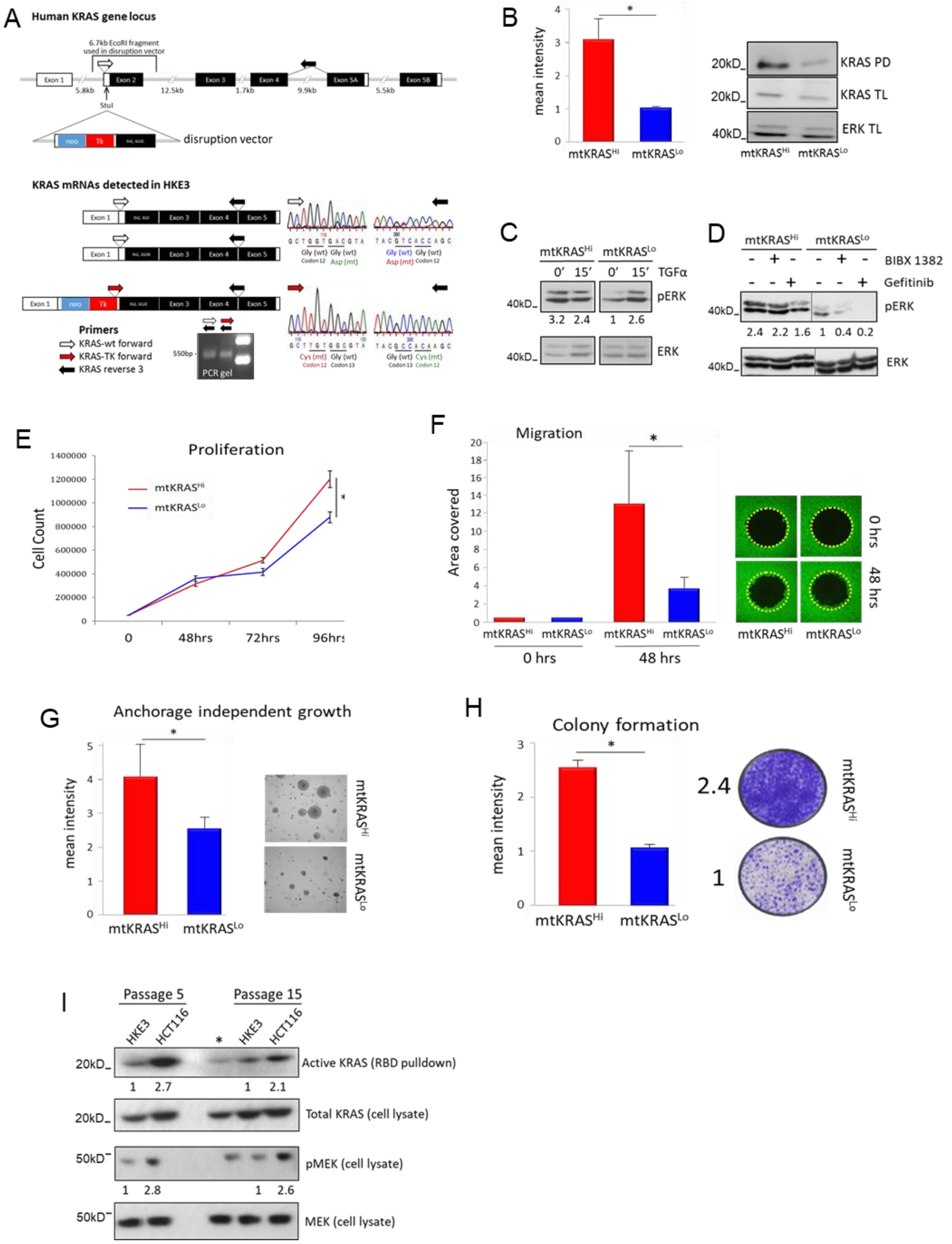

Supplementary Figure 1. Status of the *KRAS* gene, KRAS protein activity and biological characteristics of the HCT116 (mtKRAS^{Hi}) and HKE3 (mtKRAS^{Lo}) cell lines.

(A) Analysis of the KRAS gene locus in HKE3 cells. According to our whole genome sequencing and previous data [1] HCT116 cells have a duplicated mtKRAS allele. The disruption vector used to generate HKE3 cells replaces exon 2 of the endogenous KRAS gene [2]. Transcripts encoded by the KRAS genes in HKE3 were reverse transcribed, PCR amplified with the indicated primers and Sanger sequenced. The results show that HKE3 express 3 types of KRAS mRNAs: a wildtype, a G13D mutated, and one containing the G12C mutation of the disruption vector. The primers for the latter PCR product map into the Tk gene in the disruption vector and the exon 4-5 boundary of KRAS. Hence, this 550bp PCR product only can be generated if the disruption vector is correctly inserted into a KRAS gene. These results suggest that HKE3 cells contain a wtKRAS, a mtKRASG13D and a disrupted KRAS gene.

(B) Activity of KRAS proteins measured by RAS Binding Domain (RBD) pulldown assays. The results are the average of 4 independent experiments. Western blots were developed with KRAS specific antibodies and antibodies against a loading control (ERK) and quantitated using the Image J software. PD, KRAS pulldown; TL, total lysate. Error bars indicate standard deviation (SD).

(C) ERK activation in mtKRAS^{Hi} versus mtKRAS^{Lo} cells. Serum starved cells were treated with 100pg/ml TGFα. Activated ERK was determined by Western blotting with phospho-ERK specific antibodies (pERK). Blots were quantitated, and pERK values normalized to total ERK expression. Numbers below lanes represent normalized pERK.

(D) Growing cells were treated with 5µM EGFR inhibitors (BIBX1382, gefitinib) and evaluated as in C. Samples were separated on the same gel, but irrelevant lanes were removed as indicated by the dashed line. Numbers below lanes represent normalized pERK.

(E) Proliferation. 50,000 cells were seeded and counted at 48, 72 and 96 hours. Cells were cultured in DMEM containing 10% fetal calf serum (FCS) for the first 24 hours. Then, FCS was reduced to 1% to assess the ability to proliferate under reduced growth factor supply.

(F) Migration. Cells were seeded in 96-well plates with a 2mm diameter stopper in the middle. When cells reached confluency, the stopper was removed and the migration of the cells into the void was quantified.

(G) Soft agar assay measuring anchorage independent growth.

(H) Colony forming assay measuring the ability to survive and proliferate as single cells.

(I) Differences in KRAS activity between HCT116 and HKE3 cells are stable over multiple passages. Cells were analyzed at passage 5 and 15. KRAS activity was determined by an RBD-pulldown assay (27). MEK activity was detected using an antibody against the activating phosphorylation site (pMEK). Bands were quantified, and the normalized ratios (HKE3 P5 was set to 1) between active proteins and proteins expressed in the lysate are shown below the bands. The asterisk indicates a cell line that was loaded as control.

All experiments in this figure were performed in at least triplicates. Error bars represent standard deviation, and *P* values were determined by two-tailed Student's *t*-test; *, $P < 0.05$. Assays were evaluated and quantitated by Image J (B-D, F-I). Source data are provided as a Source Data file.

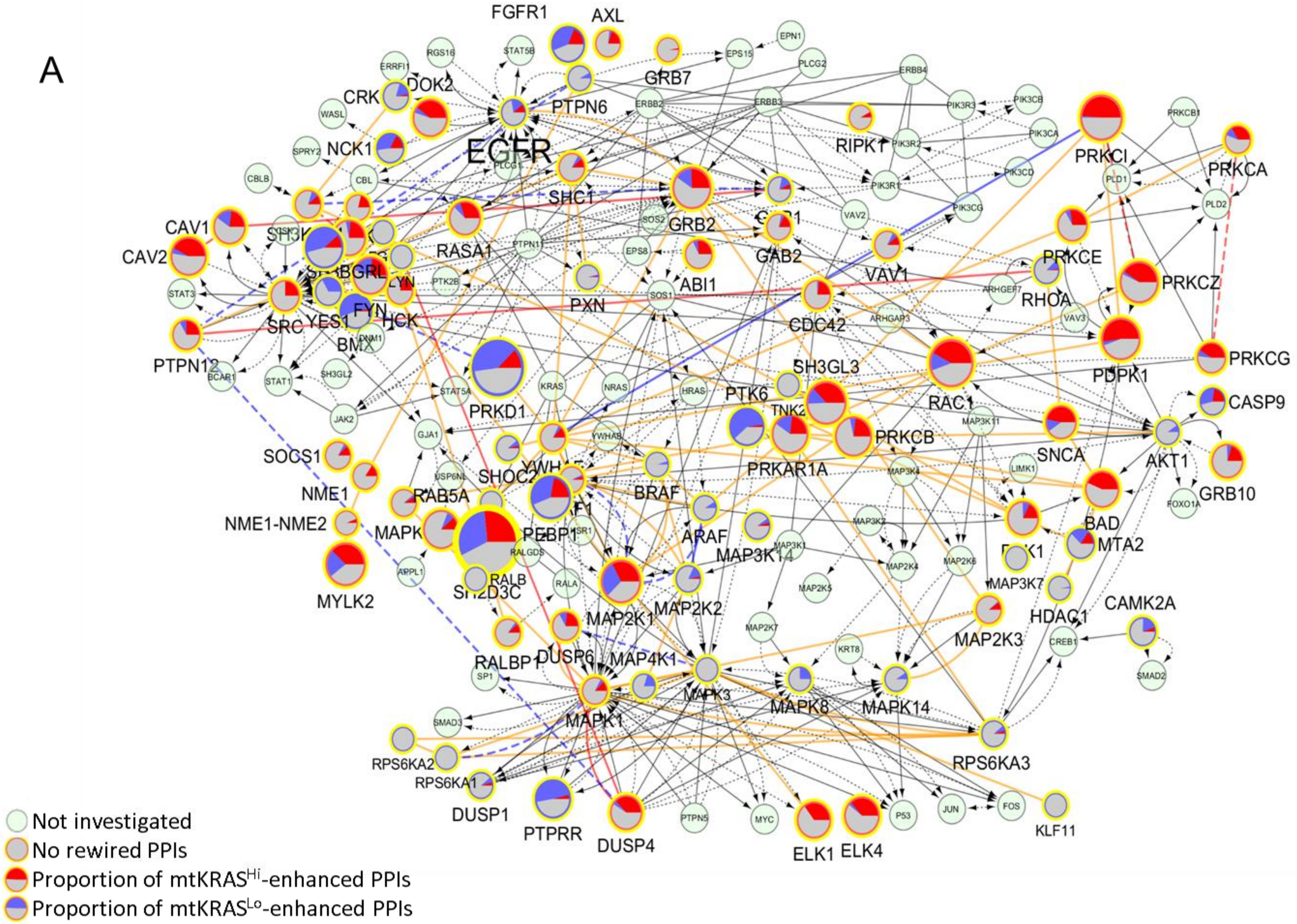
A
EGFR
FGFR1
AXL
GRB7
PTPN6
DOK2
CRK
NCK1
SHC1
GRB2
GAB2
RIPK1
PRKCI
PRKCA
PRKCE
PRKCZ
RHOA
VAV1
CAV1
CAV2
RASA1
ABI1
PXN
CDC42
SRC
YES1
FYN
HCK
LYN
BMX
PTPN12
PRKD1
SH3GL3
PTK6
TNK2
RAC1
PDPK1
PRKCG
CASP9
PRKCB
PRKAR1A
SNCA
AKT1
GRB10
SOCS1
NME1
NME1-NME2
RAB5A
SHOC2
BRAF
ARAF
MAP3K14
BAD
MTA2
MAP3K7
HDAC1
CAMK2A
MYLK2
RALB
MAP2K1
MAP2K2
MAP2K3
RALBP1
DUSP6
MAP4K1
MAPK3
MAPK8
MAPK14
MAPK1
RPS6KA3
RPS6KA2
RPS6KA1
DUSP1
PTPRR
DUSP4
ELK1
ELK4
KLF11
Not investigated
No rewired PPIs
Proportion of mtKRAS^Hi-enhanced PPIs
Proportion of mtKRAS^Lo-enhanced PPIs

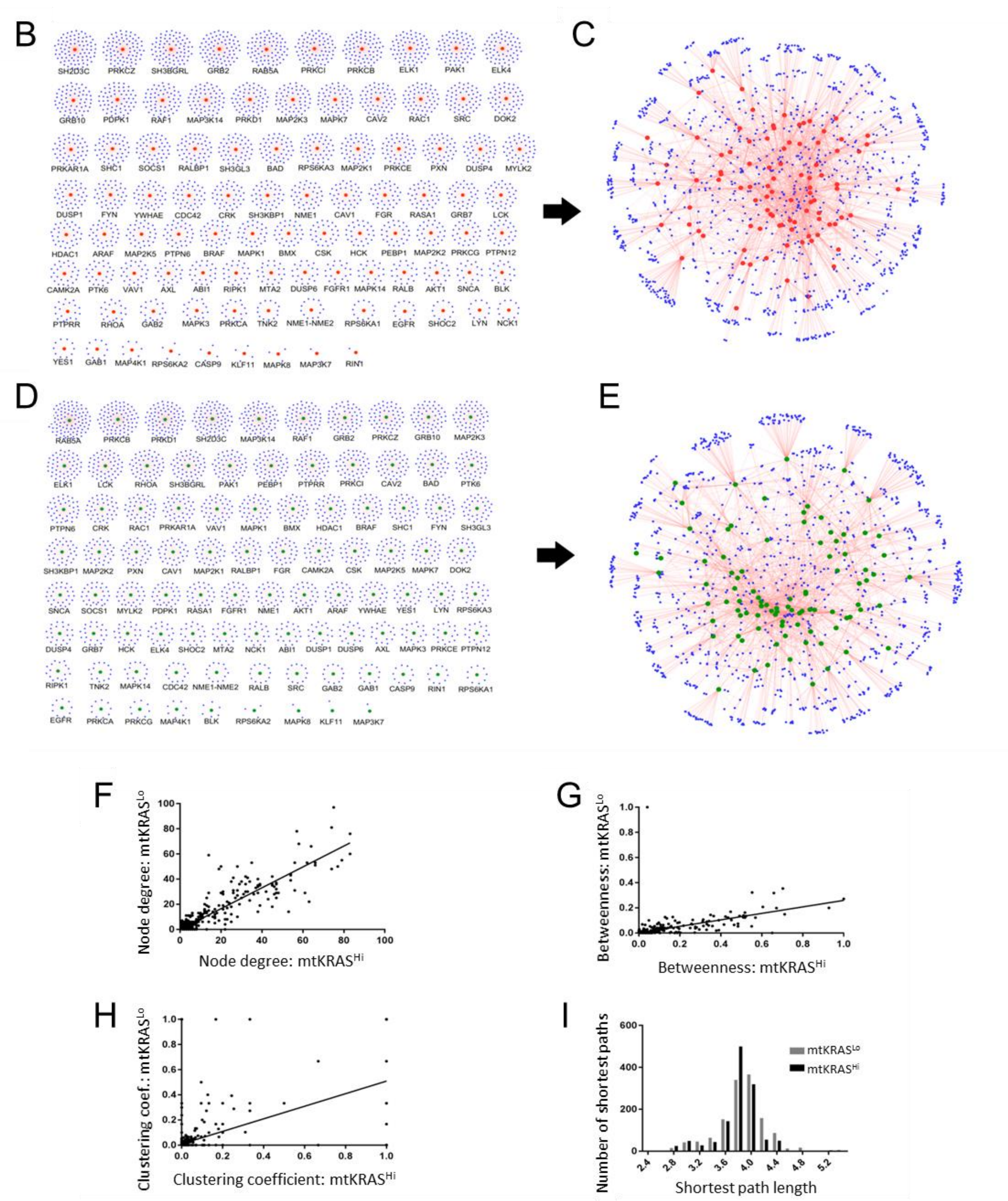

Supplementary Figure 2. The global EGFRNet$^{mtKRAS\text{-}Hi}$ and EGFRNet$^{mtKRAS\text{-}Lo}$ protein-protein interaction networks (PPINs).

(A) Summary of the EGFRNet. The 95 baits were pie-charted to show the proportions of interactions enhanced in mtKRASHi (red) and mtKRAS^{Lo} (blue); unchanged interactions are in grey. The size of the nodes is proportional to the total number of rewired interactions.

(B) Bait-prey interactions detected in mtKRAS^{Hi} cells in comparison to empty vector controls. Bait proteins are shown as red nodes, prey proteins as blue nodes.

(C) Bait-prey interactions were integrated to construct the EGFRNet$^{mtKRAS\text{-}Hi}$ PPIN.

(D) Bait-prey interactions detected in mtKRAS^{Lo} cells in comparison to empty vector controls. Bait proteins are shown as blue nodes, prey proteins as blue nodes.
(E) The EGFRNet$^{mtKRAS\text{-}Lo}$ PPIN,
(F) Node degree,
(G) node betweenness centrality,
(H) node clustering coefficient, and
(I) the distribution of the shortest path lengths were highly correlated between the two EGFRNets.

Source data are provided as a Source Data file. The Cytoscape session file for Supplementary Figure 2A is provided in Supplementary Software 2.

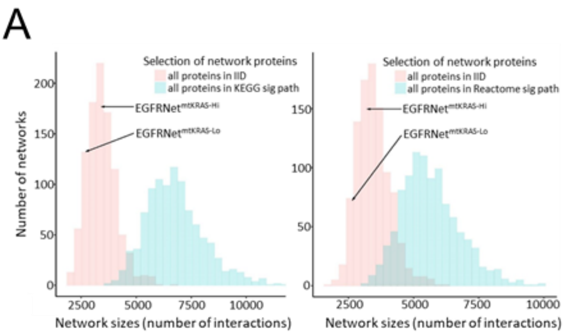

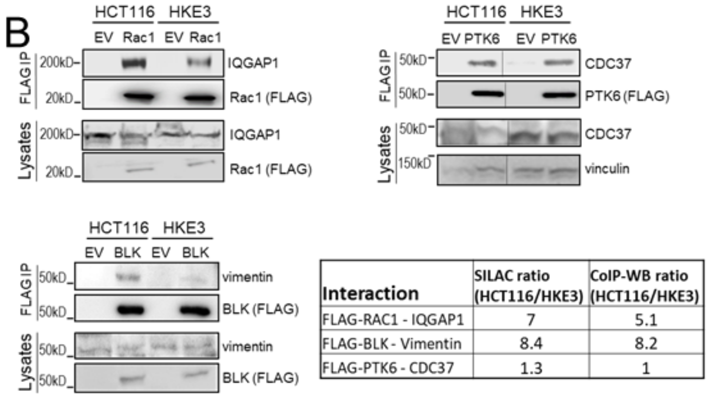

Interaction	SILAC ratio (HCT116/HKE3)	CoIP-WB ratio (HCT116/HKE3)
FLAG-RAC1 - IQGAP1	7	5.1
FLAG-BLK - Vimentin	8.4	8.2
FLAG-PTK6 - CDC37	1.3	1

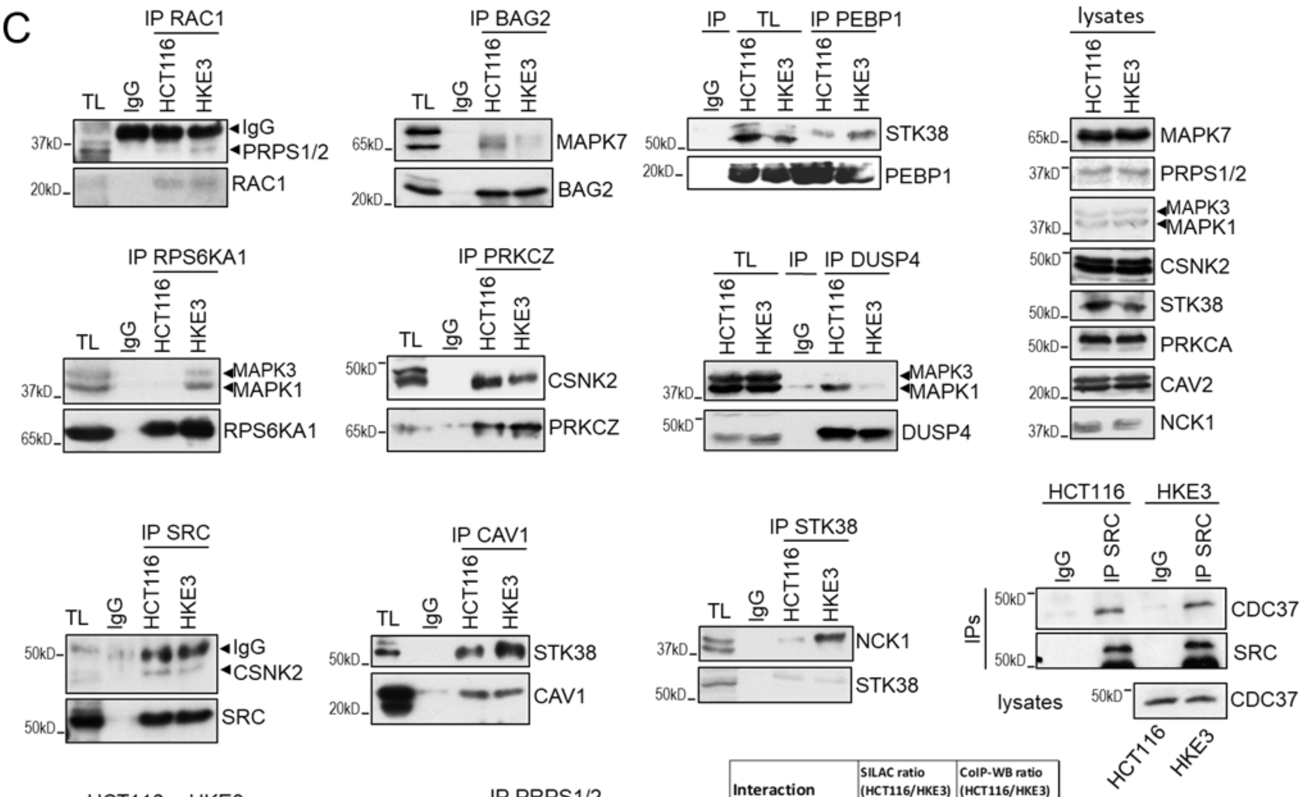

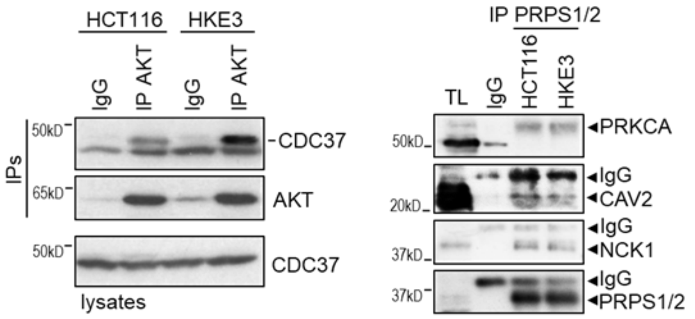

Interaction	SILAC ratio (HCT116/HKE3)	CoIP-WB ratio (HCT116/HKE3)
RAC1 - PRPS1/2	0.4/0.4	0.5
MAPK7 - BAG2	1.7	2.4
SRC - CSNK2A1	1.4	1.4
NCK1 - PRPS1	5.2	1.3
PRKCA - PRPS2	1.3	1.1
CAV2 - PRPS1/2	1.4/1.5	1.7
STK38 - NCK1	0.31	0.2
PRKCZ - CSNK2A1/2B	1.9/1.7	2
RPS6KA1 - MAPK1	0.6	0.4
CAV1 - STK38	0.3	0.4
DUSP4 - MAPK1	4	4.6
PEBP1 - STK38	0.3	0.28
AKT1 - CDC37	0.5	0.5
SRC - CDC37	1	0.9

Supplementary Figure 3. Expected network size distributions for 93 bait proteins.
(A) Expected size distribution of the EGFRNets. 93 bait proteins were identified to have at least one interaction in $EGFRNet^{mtKRAS-Hi}$ and $EGFRNet^{mtKRAS-Lo}$. The pink distributions show the expected numbers of experimentally validated interactions for 93 randomly sampled proteins in the Integrated Interaction Database (IID) version 2018-11[3]. Proteins and their interactions were randomly sampled from IID 1000 times to construct these distributions. The numbers of interactions detected in the $EGFRNet^{mtKRAS-Hi}$ and $EGFRNet^{mtKRAS-Lo}$ PPINs fall close to the middle of these distributions. Since bait proteins were not a random sample, but rather known members of the EGFR signaling pathway, network size distributions were also generated for sets of signaling proteins. The green distributions show the expected numbers of experimentally validated interactions for 93 randomly sampled proteins from KEGG (left panel) and Reactome (right panel) signaling pathways. KEGG and Reactome signaling pathway proteins and their interactions were randomly sampled from IID 1000 times to construct these distributions. Networks of signaling pathway proteins were considerably larger than those of randomly selected proteins; mean network sizes were 6,657 PPIs for KEGG signaling pathway proteins, 5,656 PPIs for Reactome signaling pathway proteins, and 3,459 for randomly selected proteins. Signaling-related networks were larger because proteins annotated to signaling pathways have been studied more extensively; they have been tested in more PPI screens than random proteins and their PPI networks have accumulated more interactions. Consequently, the two EGFRNets were similar in size to random protein networks, and considerably smaller than signaling-related networks, which likely represent many more screens.
(B) Validation of qMS results by co-immunoprecipitation (co-IP)/Western blotting (WB). HCT116 and HKE3 cells were transfected with the indicated FLAG-tagged baits. Cells were grown to confluency and serum deprived for 20 hours before being lysed and immunoprecipitated using anti-FLAG. Western blots were stained with the antibodies indicated. The PTK6-CDC37 co-IPs were on the same blots, but lanes were spliced together as indicated by broken lines. EV, empty vector transfection.
(C) Endogenous co-IP/WB experiments were carried out using the indicated antibodies. WBs were scanned and quantitated using Image J. Signals corresponding to co-immunoprecipitated prey proteins were normalized to the amount of pulled-down bait and expression of the prey in total cell lysates. These ratios were compared to qMS data. In most cases the qMS and co-IP/WB ratios corresponded well except in one case (NCK1-PRPS1) shown in red.

Source data are provided as a Source Data file. The R-code and source data for Supplementary Figure 3A are provided in Supplementary Software 3.

A

B

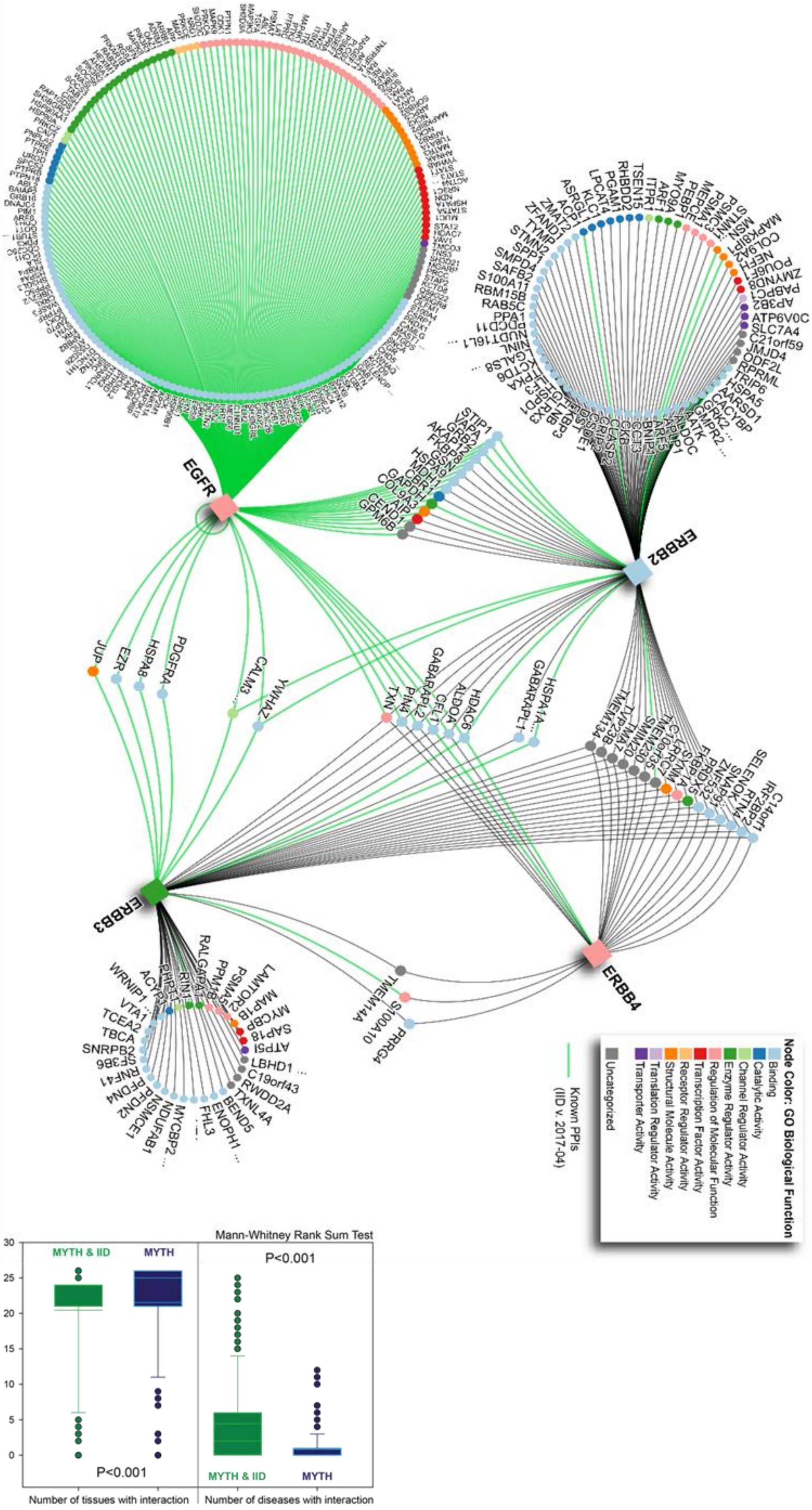
EGFR
ERBB2
ERBB3
ERBB4
Node Color: GO Biological Function
Binding
Catalytic Activity
Channel Regulator Activity
Enzyme Regulator Activity
Regulation of Molecular Function
Transcription Factor Activity
Receptor Regulator Activity
Structural Molecule Activity
Translation Regulator Activity
Transporter Activity
Uncategorized
Known PPIs
(IID v. 2017-04)
Mann-Whitney Rank Sum Test
MYTH & IID
MYTH
P<0.001
P<0.001
Number of tissues with interaction
Number of diseases with interaction

Supplementary Figure 4. The ERBB1-4 protein interaction network. (A) MYTH was used to identify binary protein interactors of the human ERBB2, ERBB3, and ERBB4 proteins. These data were combined with known MYTH-based interactions for ERBB1-4 from the IID database [4] to construct the network shown featuring 405 interactions (Supplementary Data 3). The network was visualized using NAViGaTOR 3 [5]. The ERBB2-4 interactome comprises 197 interactions, of which 181 are new. Twenty-nine proteins interact with more than one ERBB receptor, and according to GO analysis 97 preys (~25%) are membrane proteins. GO molecular function terms were used to colour nodes, as per inset legend. Blue edges indicate interactions that were previously detected by MYTH in other studies. **(B)** The ERBB family interactomes (Supplementary Data 3) show a significant (P<001; Mann-Whitney Rank Sum Test) bias both in tissue, and disease distributions for MYTH interactions supported by other experimental data or predicted evidence from IID. Highly tissue specific are ITPKA-EGFR, LRRC7-ERRB3, LRRC7-ERBB4, CDC25C-EGFR, and ISK-EGFR. Highest disease annotation is available for EGFR-EGFR, STAT3-EGFR, CRK-EGFR, and AKT1-EGFR.

Source data are provided as a Source Data file.

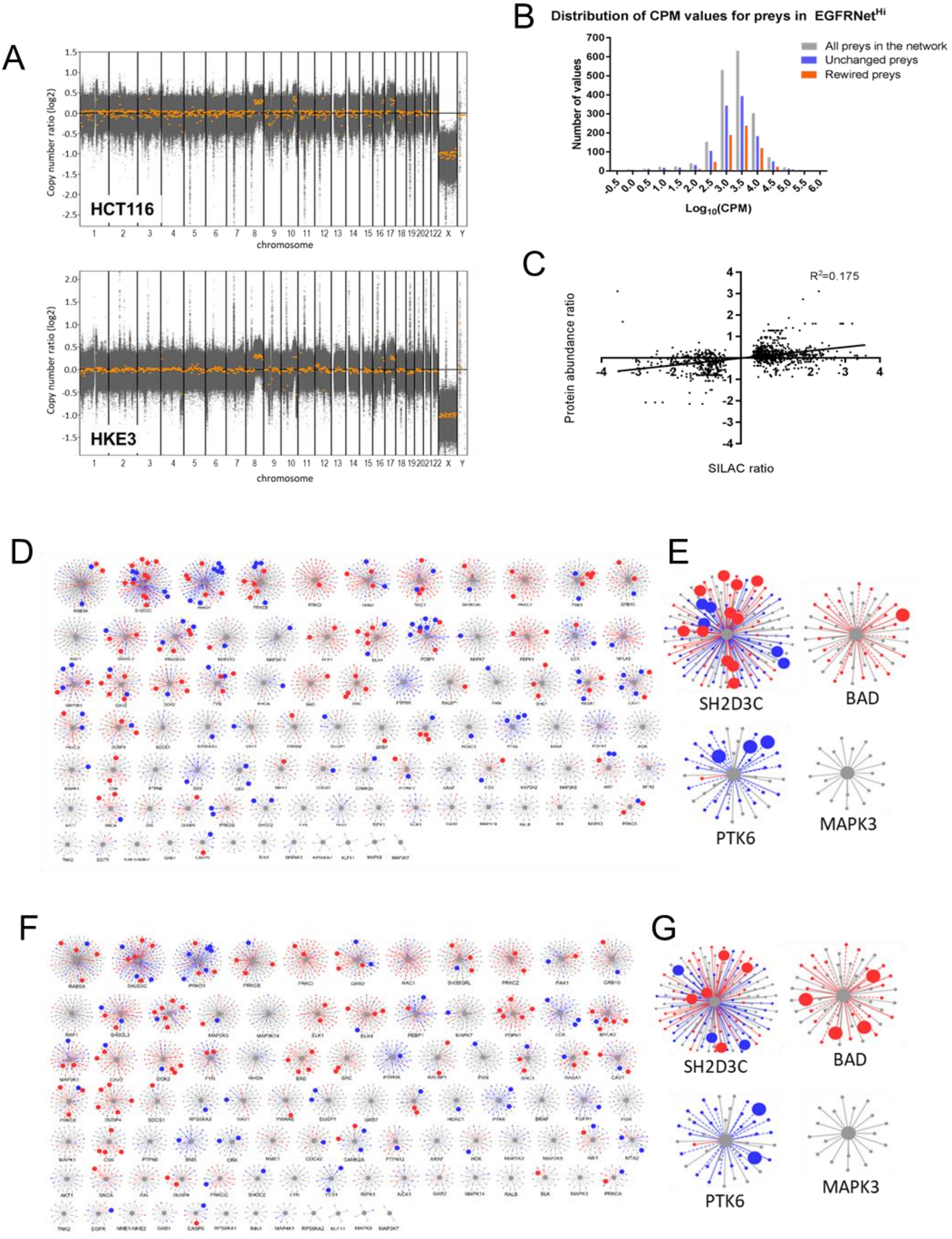
A
Copy number ratio (log2)
HCT116
chromosome
HKE3
B
Distribution of CPM values for preys in EGFRNet^Hi
All preys in the network
Unchanged preys
Rewired preys
Number of values
Log10(CPM)
C
R2=0.175
Protein abundance ratio
SILAC ratio
D
E
SH2D3C
BAD
PTK6
MAPK3
F
G
SH2D3C
BAD
PTK6
MAPK3

Supplementary Figure 5. Potential drivers of EGFRNet$^{mtKRAS-Hi}$ and EGFRNet$^{mtKRAS-Lo}$ PPI network rewiring.

(A) Copy number variation analysis.

(B) RNAseq data showing that the distribution of gene expression values was similar mtKRAS^{Hi} or mtKRAS^{Lo} cell lines for unchanged and rewired preys, i.e. there was no bias towards higher or lower gene expression for the rewired preys.

(C) There was a weak ($r^2 = 0.18$) but significant correlation ($P < 0.001$) between fold-change in abundance of proteins in the IPs as determined by AP-MS and fold-change in protein expression between the two cell lines.

(D) Rewired interactions involving differentially expressed preys. Proteins that were significantly differentially expressed between the mtKRAS^{Hi} and mtKRAS^{Lo} cell lines were mapped to bait-prey interactions detected in EGFRNet$^{mtKRAS-Hi}$ and EGFRNet$^{mtKRAS-Lo}$. Interactions enhanced in EGFRNet$^{mtKRAS-Hi}$ and EGFRNet$^{mtKRAS-Lo}$ are shown in red and blue, respectively. Differentially expressed proteins are indicated by larger sized nodes.

(E) Four selected AP-MS complexes highlighting differentially abundant prey proteins (larger nodes) are also shown.

(F) Rewired interactions involving differentially phosphorylated preys. Differentially phosphorylated proteins were mapped to bait-prey interactions in EGFRNet$^{mtKRAS-Hi}$ and EGFRNet$^{mtKRAS-Lo}$. Color scheme as in A, with differentially phosphorylated proteins shown as larger sized nodes.

(G) Four selected AP-MS complexes highlighting differentially phosphorylated prey proteins (larger nodes) are shown.

Source data are provided as a Source Data file. The Cytoscape session files for Supplementary Figures 5C,D is provided in Supplementary Software 4, and for Figures 5E,F in Supplementary Software 5.

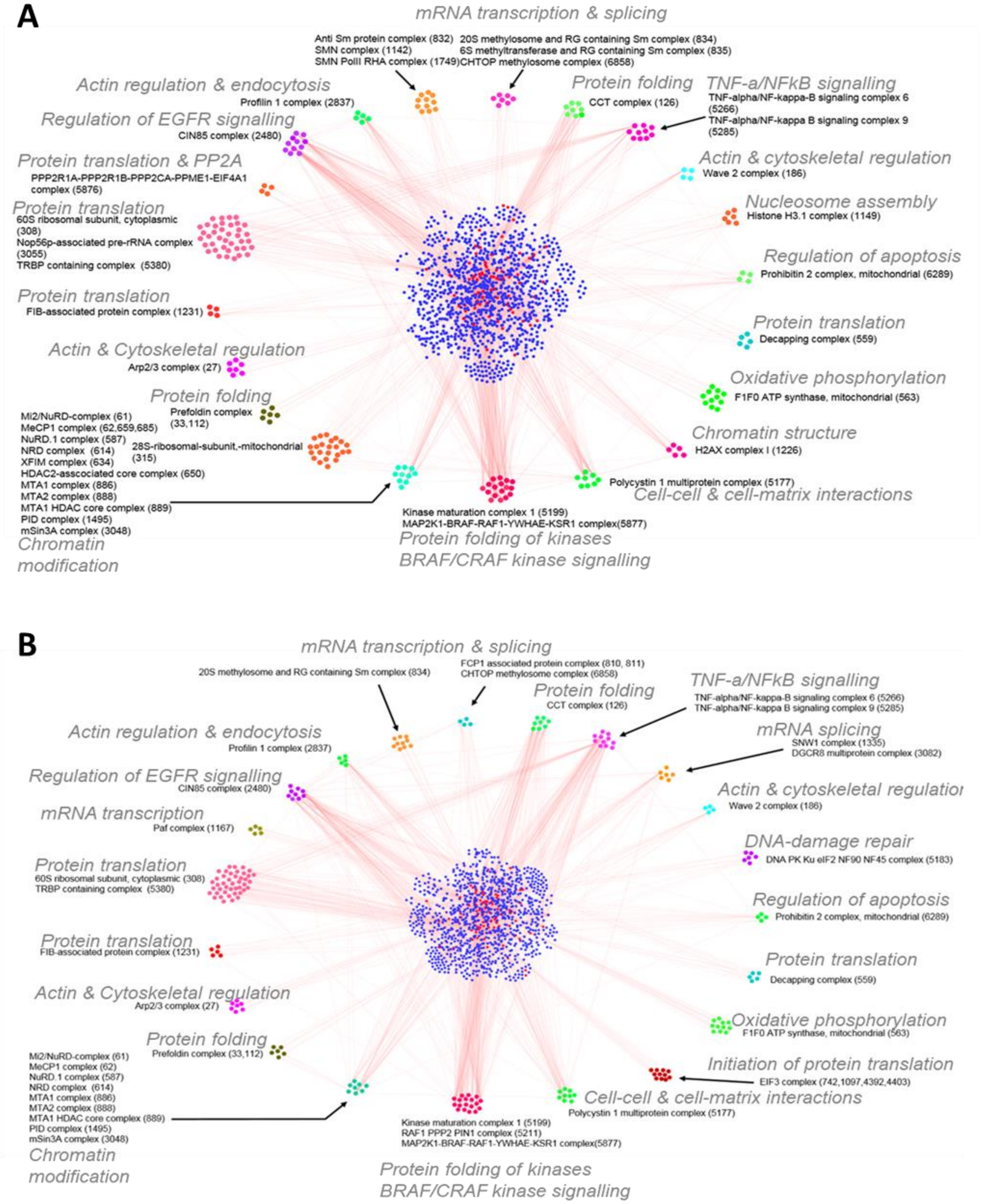

Supplementary Figure 6. CORUM complexes identified in the (A) EGFRNet$^{mtKRAS-Hi}$ and (B) EGFRNet$^{mtKRAS-Lo}$ PPINs. CORUM complexes are shown, where at least 70% of the complex component proteins were nodes in the EGFRNets. Nodes annotated in the same CORUM complex are in the same colour. In some cases, multiple complexes map to an overlapping set of nodes in the network. Bait proteins are shown as red nodes. Prey proteins not annotated in the identified CORUM complexes are shown as blue nodes. CORUM complex ID numbers are in parenthesis. Source data are provided as a Source Data file. The Cytoscape session file for Supplementary Figure 6 is provided in Supplementary Software 6.

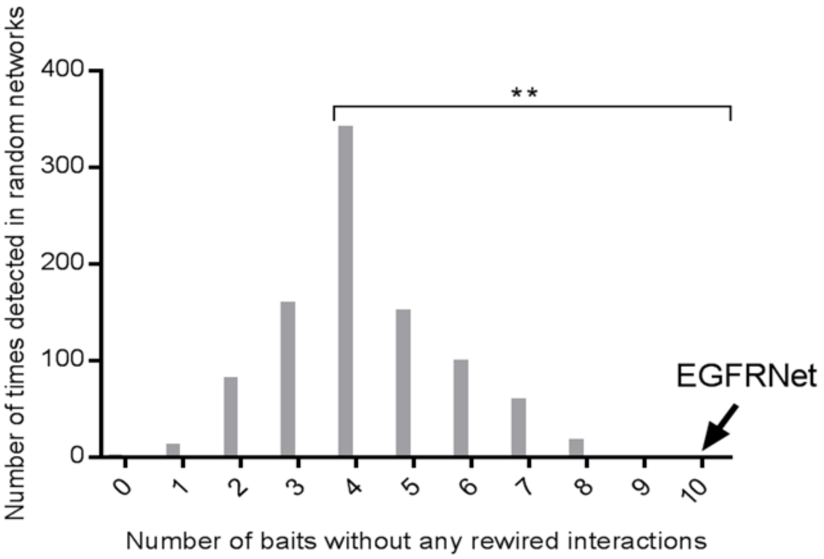

Supplementary Figure 7. Rewired interactions are non-randomly distributed among baits. To determine whether rewired interactions were non-randomly distributed among the 93 bait proteins, edge labels were permuted 1,000 times and the number of baits with no rewired interactions assessed in each simulation. The number of baits with no rewired interactions observed in the EGFRNets (10 baits) was significantly (P<0.002) outside the random distribution as indicated by **. Statistical significance was assessed using the Kolmogorov–Smirnov test.

Source data are provided as a Source Data file.

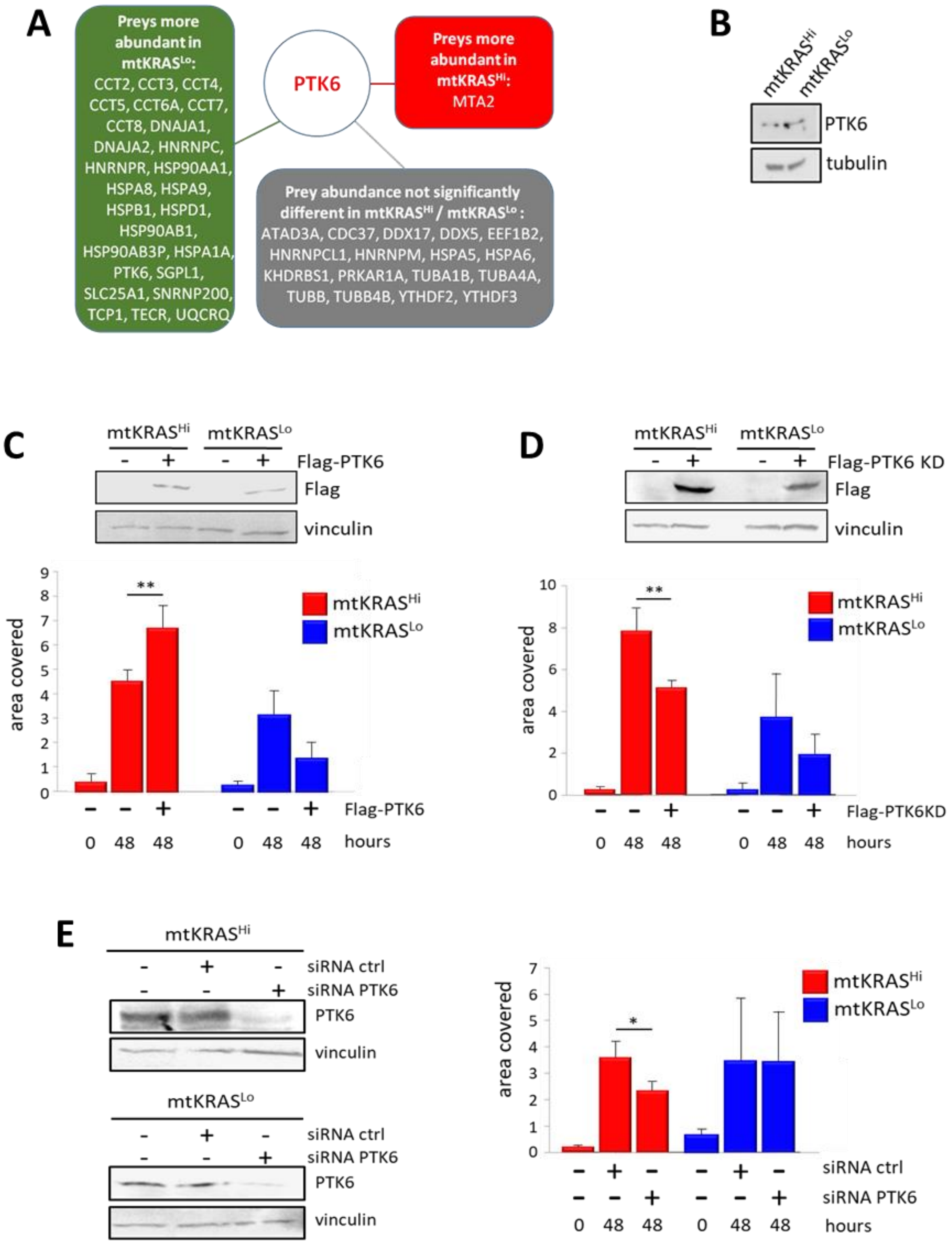

Supplementary Figure 8. PTK6 regulates cell migration.
(A) Differential PTK6 complex composition.
(B) Western blot showing similar expression of endogenous PTK6 in mtKRAS^{Hi} and mtKRAS^{Lo} cells. Tubulin was used as loading control.
(C) FLAG-PTK6 was transfected into mtKRAS^{Hi} and mtKRAS^{Lo} cells, and migration was measured using the Oris^{TM} 2D Cell Migration Assay. FLAG-PTK6 expression was assessed

by Western blotting with anti-Flag, vinculin was used as loading control.
(D) Expression of the PTK6 K219M kinase dead (KD) mutant significantly decreased cell migration in mtKRAS^{Hi} cells.
(E) PTK6 knockdown by siRNA (smartpool, Dharmacon) reduced cell migration specifically in mtKRAS^{Hi}. A non-targeting siRNA was used as control, and knockdown was ascertained by Western blotting of endogenous PTK6. Error bars represent standard deviation, and *P* values were determined by two-tailed Student's *t*-test; *, $P < 0.05$; **, $P < 0.01$; differences without *P* values indicated were not significant.
Source data are provided as a Source Data file.

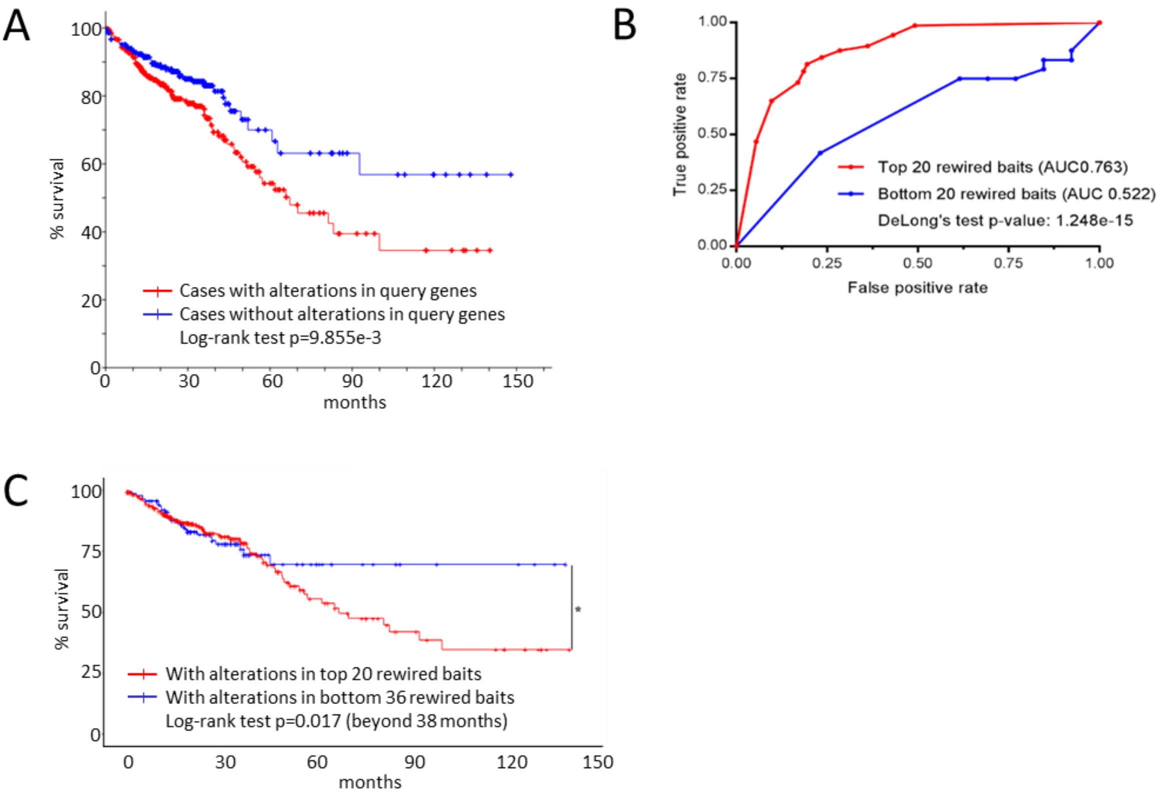

Supplementary Figure 9. Clinical correlates of PPIN rewiring.
(A) CRC patient overall survival curves ranked by the baits that have the most enhanced interactions in the mtKRAS^{Hi} PPIN.
(B) To assess the accuracy of the top-20 bait proteins to classify patients into high and low risk groups, we trained a Lasso classifier using RNAseq expression, copy-number, and mutation data from TCGA patients. Five-fold cross-validation of the classifier by subsampling the patient data into training (80%) and testing (20%) datasets gave an accuracy of up to 0.79 (mean 0.70) and an area under the ROC curve (AUC) of 0.763 (red curve). A similar classification using the bottom-20 rewired proteins (blue curve) gave a much lower mean accuracy of 0.4 and AUC of 0.522.
(C) Kaplan-Meier survival curves for TCGA CRC patients with alterations in the top 20 rewired bait proteins (red) compared to patients with alterations in the bottom 36 least rewired proteins (blue). The log-rank test was used to assess statistical significance; * $P < 0.05$.
Source data are provided as a Source Data file. The R-script for Supplementary Figure 9B is provided in Supplementary Software 7.

Supplementary Methods

1. Cell lines and cell culture. HCT116 (mtKRAS^{Hi}) and HKE3 (mtKRAS^{Lo}) cells [2] were provided by Doctors Shirasawa and Sasazuki. All cells were grown in DMEM supplemented with 10% fetal calf serum (FCS) and 1% L-glutamine (Gibco-BRL). Cells were routinely tested for mycoplasma contamination. Cell lines were authenticated by RNAseq as recently described [6].

In order to test whether the cells were clonal populations we used fluorescence-activated cell sorting to isolate ~1,000 single cells from the HCT116 and HKE3 cell lines and seeded them into 96 well plates. Once colonies were visible, cells were treated with 1mM G418 or 1mM Ganciclovir (GCV). The gene targeting vector used by Shirasawa and colleagues carries a neomycin gene conferring resistance to G418, and a thymidine kinase gene conferring sensitivity to GCV [2]. Thus, cells carrying the targeting vector are G418 resistant and GCV sensitive, whereas cells lacking the targeting vector are G418 sensitive and GCV resistant. This was the pattern observed without exception. All HKE3 clones were resistant to G418, but died in GCV medium, whereas all HCT116 clones were killed by G418, but grew in the presence of GCV. This experimental setup would have detected a contamination of HKE3 by HCT116 down to a level of ~0.1%. Thus, these results confirmed that the cells are not cross-contaminated by clones from the other cell line.

Whole genome sequencing (WGS) and RNA expression sequencing (RNAseq) of HCT116 and HKE3 was used to further investigate why mtKRAS^{G13D} is still expressed in HKE3 cells. The WGS data showed a 1:1.6 allele ratio of wt/mt KRAS in HCT116 cells indicating that HCT116 may contain a duplicated mutant KRAS allele (Supplementary Table 1). This is consistent with a previous report showing duplication of the mtKRAS allele in HCT116 cells [1]. The presence of the disruption vector in the HKE3 genome was confirmed by finding the KRAS G12C mutation, which is contained in the disruption vector used to knock-out the mutant KRAS G13D allele. In the event of a successful disruption, the inserted Neo-Tk cassette produces a mRNA but cannot encode a protein, as protein translation will terminate after the Tk gene [2]. The KRASG12C mutation also is detected at the mRNA level further confirming that the disruption cassette is integrated in the HKE3 genome.

	KRAS gene status	G12G + G13G (GGT GGC) Wildtype (wt)	G12G + G13D (GGT GAC) Mutant (mt)	G12C + G13G (TGT GGC) dv & wt	G12C + G13D (TGT GAC) dv & mt
DNA	HCT116	12 (1)	19 (1.6)	0 (0)	0 (0)
	HKE3	14 (1)	12 (0.9)	15 (1.1)	0 (0)
RNA	HCT116	45 (1)	37 (0.8)	0 (0)	0 (0)
	HKE3	50 (1)	51 (1)	37 (0.7)	0 (0)

Supplementary Table 1. *Comparison of WGS and RNAseq results between HCT116 and HKE3 cells. dv, disruption vector. Ratios (within a row) are given in parentheses.*

In order to examine whether the disruption vector has integrated into the KRAS locus we analysed the KRAS mRNAs expressed in HKE3 cells by PCR (Supplementary Figure 1A). PCR was performed on cDNA from HKE3 using two primer combinations: (i) a forward primer (open arrow) that binds to the endogenous KRAS sequence just upstream of the StuI restriction site, where the Neo-Tk cassette in the disruption vector was inserted; plus a reverse primer (black arrow) that binds to the exon 4-5 junction well outside of the disruption vector; and (ii) a forward primer (red arrow) that binds to the Tk sequence; plus the same reverse primer (black arrow). Both primer combinations produced PCR products of the expected size (ca. 550bp).

The combination of the Tk primer and the KRAS exon 4/5 boundary primer only can generate this product, if the disruption vector is correctly integrated at the KRAS gene locus. Sanger sequencing of the PCR products showed that HKE3 cells contain three types of mRNA transcripts, i.e. wt KRAS mRNA, KRASG13D mRNA, and KRASG12C mRNA, which was only detected when the primer binding the Tk gene was used. The presence of these 3 types of KRAS transcripts indicates that HKE3 have a wt KRAS gene, a mt KRAS G13D gene, and a disrupted mt KRAS gene, where the endogenous G13D mutation was replaced by the G12C mutation carried by the disruption vectors. This result is consistent with the observation that HCT116 cells have two mt KRAS G13D alleles (see above), and that the disruption vector only has disrupted one mt KRAS allele while leaving the second intact. This finding also explains why HKE3 express a reduced amount of KRAS G13D mRNA and protein as compared to HCT116 cells.

This interpretation is also consistent with the circa threefold difference in KRAS activity between HCT116 and HKE3, which has remained constant over at least 10 continuous passages (Supplementary Figure 1I). This difference in KRAS activity also was stable during a >3 year period of intermittent culturing these cell lines in our laboratories (compare Supplementary Figure 1B in the current manuscript with Fig. 5 in Fasterius et al. [6]. If the HKE3 population had contained a mtKRAS clone, it would have expanded in this period of time. Based on the difference in proliferation rates (Supplementary Figure 1E), a mtKRAS clone or a contaminating HCT116 cell would have completely taken over the HKE3 population within 8 months when present at an initial 1 : 1 million ratio. This would have profoundly shifted the antibiotic resistance measured in single cells (see above), the KRAS allele ratio, the biochemically measured KRAS activity ratio, and the biological behaviour. As neither of these changes was observed, we conclude that the low expression of $mtKRAS^{G13D}$ in the HKE3 cells is not due to contamination with HCT116 or a back-mutated clone in the HKE3 population, and that we are comparing a population of isogenic cells that express high ($mtKRAS^{Hi}$) and low levels ($mtKRAS^{Lo}$) of $mtKRAS^{G13D}$.

<u>2. PCR analysis.</u> Cells were grown to 80% confluence and lysed directly in the dish using 600µL RLT Plus buffer (Qiagen) after removal of the growth media. RNA was isolated with the RNeasy Plus Mini Kit (Qiagen), transcribed into cDNA using the SuperScript III First Strand Synthesis System (ThermoFisher Scientific) with oligo(dT) primers, and then amplified by PCR using Dynazyme II DNA Polymerase (ThermoFisher Scientific) according to the manufacturer's protocol with an annealing temperature of 55°C. Two sets of primer pairs were used (i) KRAS-TK forward + KRAS reverse 3; (ii) KRAS-wt forward + KRAS reverse 3. The PCR product was purified using QIAquick PCR cleanup (Qiagen) and Sanger sequenced by Eurofin Genomics.

Primers sequences:
KRAS-TK forward: 5'-CTGGTACGAGGAGCGCTTT-3'
KRAS-wt forward: 5'-GACTGAATATAAACTTGTGGTAGTTGG-3'
KRAS reverse 3: 5'-ACACCCTGTCTTGTCTTTGCT-3'

<u>3. Whole genome sequencing.</u> DNA extractions were performed with the DNeasy Blood and Tissue Kit (Qiagen) as per the manufacturer's instructions. Extracted nucleic acids were stored at -80°C before sequencing. Whole genome sequencing library preparation was performed with the TruSeq DNA PCR-free Kit (Illumina) and sequenced on a HiSeqX instrument. The quality assessment of the WGS data is shown in Supplementary Table 2. The genome sequencing data

have been submitted to the NCBI sequence read archive under accession number PRJNA374513 (https://www.ncbi.nlm.nih.gov/sra/?term=PRJNA374513).

	**HCT116**	**HKE3**
**Total Reads**	628,300,083	644,210,762
**Number of aligned Reads**	625,454,093	641,060,604
**Aligned Reads (percentage of total reads)**	99.55%	99.51%
**Average Autosomal Coverage**	30.66X	31.10X
**GC Content 40.39%**	40.79	40.39

Supplementary Table 2. *Quality assessment of WGS of HCT116 and HKE3 cells.*

4. Single Nucleotide Variant (SNV) and small insertion-deletion (InDel) site analysis. WGS reads were aligned to the GRCh37 human reference genome using *bwa mem* [7]. Variants were called using Genome Analysis Toolkit (GATK v4.1.30) pipelines. Briefly, the GATK HaplotypeCaller was used for calling single nucleotide polymorphisms (SNPs) and InDels, followed by the GenotypeGVCF (genomic variant call formats) for joint genotyping of the HCT116 and HKE3 cells [8]. SNVs were filtered separately for SNPs and InDels using the GATK variant quality score recalibration (VQSR) pipeline and annotated using the Ensembl (release 97) Variant Effect Predictor [9]. After filtering, a total of 170,135 SNVs and small InDels with 10 or more supporting reads were identified between HKE3 and HCT116. Of these discordant variants, 1,091 were found within sites defined as having a variant of “high” or “medium” impact (Supplementary Table 3) defined by 12 scenarios that alter protein coding sequences (https://asia.ensembl.org/info/genome/variation/prediction/predicted_data.html). In total, 872 genes were predicted to contain SNVs with high or medium impact. Of these genes, 495 were expressed on average >1 log2(reads per million) in the two cell lines. Of these, 70 were nodes in the EGFR PPI network and 36 were rewired. Considering that the EGFR PPI networks contain 4,420 nodes, of which 1360 have rewired interactions, SNVs affect 1.6% of nodes and 2.6% of rewired interactions. These data suggest that SNVs may be linked to the rewiring of a small number of EGFR network nodes, but cannot explain the extensive rewiring of 30.7% PPIs observed.

**Impact**	**Impact level**	**# sites**
missense_variant	MED	757
frameshift_variant	HIGH	159
splice_region_variant	MED	115
stop_gained	HIGH	27
splice_donor_variant	HIGH	16
splice_acceptor_variant	HIGH	14
inframe_deletion	MED	14
inframe_insertion	MED	6
start_lost	HIGH	4
protein_altering_variant	MED	1
Total		1113

Supplementary Table 3. *Annotated SNP/InDel discordant sites with HIGH and MEDIUM impact identified using Variant Effect Predictor (VEP).*

5. Copy Number (CNV) and structural variants analysis. Structural and copy number variants were called on the aligned WGS files with Manta version 1.6.0 [10] using a joint diploid sample

workflow (i.e. without tumour-normal matching) and cnvkit version 0.9.5 [11]. After removing sites that did not pass quality filters and problem regions such as centromeric/telomeric and low-complexity regions previously identified in [12], discordant structural variant were then extracted using *bcftools* and annotated using ANNOVAR [13]. 476 discordant structural variants were detected between HKE3 and HCT116, 14 of which contained one or more annotated genes. Of these, all were variable in HKE3 cell line and homozygous reference in the HCT116. The mean size of these variants was 733,262bp. In total, 27 genes were predicted to be impacted by structural variants. Of these genes, only 12 were expressed on average >1 count per million across both cell lines and no gene was a node in the EGFR PPI network. Only 3 genes [ALPP; EREG; ZNF91], predicted to differ in their copy number between the cell lines (CNV genes), were significantly differentially expressed (FC > 2; FDR < 0.05). Only 1 of these genes [EREG] were also identified as being significantly differentially abundant at the protein level (Significance A < 0.05). As with the structural variants, CNVs were called between HKE3 and HCT116 to identify any gain or loss of gene that may impact observed network rewiring. Using a log2 weighted mean threshold of 0.4, the level of gain or loss of coverage in each sample, HCT116 contained 1688 genes in CNVs compared to 3103 for HKE3 (Supplementary Figure 5A). Removing common gene CNVs a total of 2310 genes were identified to be different between the two samples. Of these genes, 137 were expressed on average >1 count per million, 5 were found to be in network nodes and one gene, PPP3CA, was rewired. These data suggest that structural variant nor CNV-driven changes in gene/protein expression have little impact on the observed network rewiring. Unexpectedly, this analysis did not detect a CNV that includes the KRAS locus, which was suggested by the results reported in Table 1 and Supplementary Figure 1A. While this discrepancy likely is related to technical limitations of the different experimental and analyses methods, we cannot fully explain the reasons behind these results. However, despite these ambiguities of the genetic analysis, the biochemical analysis of the KRAS protein activity showed that HCT116 cells have a 3fold elevated KRAS activity over HKE3 cells (Supplementary Fig. 1B). This result was obtained by MS and Western blot analysis (Supplementary Fig. 1B) and was very stable over time (Supplementary Figure 1I; also compare Supplementary Figure 1B to Fig. 5 in Fasterius et al.[6]).

<u>6. Antibodies and reagents.</u> Antibodies were contributed by the Human Protein Atlas project, and the following antibodies were obtained from commercial sources: K-RAS (sc-30), Tub TU-02 (sc-8035), PTK6 C18 (sc-1188), PRPS1/2/L (sc-292588), CAV2 (sc-7942), CDC42 (sc-87), RSK1/ RPS6KA1 (sc-231), RKIP/PEBP1 (sc-28837), PRKCA (sc-20), PRKCZ (sc-17781), BAG2 (sc-366091), and CAV1 (sc-894) from Santa Cruz; GAPDH 1410C (2118S), Paxillin (2542S), Vinculin (4650), BAD (9292), phospho-BAD S112 (9296), phospho-AKT (9271S), AKT (9272S), NCK1 (2319), SRC (2123), MAPK7/ERK5 (3372) from Cell Signaling. ERK1/2 (M5670), phospho-ERK1/2 (M8159), anti-Flag M2 peroxidase (A8952), protein G-sepharose, anti-FLAG-M2 conjugated agarose beads (A2220), DUSP4 (SAB1403748) and Calcein (C1359) were from Sigma-Aldrich. STK38/NDR1 (BD 610828) and IQGAP1 (BD 610611) were from BD Biosciences. RAC1 (05-389) was from EMD Millipore. CSNK1/2 (ab10474) and CDC37 (ab56598) were from Abcam. BCA protein assay kit (Pierce 23225) was from Thermo Fisher Scientific. Empore ™ C18 Extraction Discs were from 3M. Gefitinib was from American Custom Chemicals Corporation, EGF receptor inhibitorII (BIBX 1382) from Calbiochem, and H-89 dihydrochloride hydrate (B1427) from Sigma-Aldrich. For Western blotting all antibodies were used at a 1:1,000 dilution with the exceptions of ERK and phospho-ERK that were diluted 1:10,000; and KRAS and RAC1 antibodies which were diluted 1:500.

7. siRNAs. ON-TARGET plus si CONTROL NON-TARGETing siRNA (D-001810-01-20), ON-TARGETplus SMARTpool PTK6 siRNA L-003166-00-0010 and ON-TARGETplus SMARTpool BAD (L-003870-00-0005) were from Dharmacon.

8. Baits and expression vectors. To achieve a high degree of coverage of the EGFR network we carried out AP-MS on 95 baits (Supplementary Data 1). These baits were selected from a previous manually-curated dataset of the EGFR network [14] and through additional literature review. The cDNAs for the bait proteins were ordered from Origene and cloned into the SF-TAP vector [15] with the tag at the N-terminus using the Gateway cloning system (Thermo Fisher) according to the manufacturer's instructions. FLAG-PTK6 KD was generated by site directed mutagenesis changing the catalytic lysine 219 to methionine.

9. SILAC labelling and bait protein isolation**.** Expression efficiency of the different cDNAs was determined by introducing increasing amounts of the plasmids into the SILAC labelled cell lines using polyethylenimine (408727, Sigma Aldrich) transfection as described [16]. Relative bait protein expression in the two cell lines was determined by Western blotting. Subsequently, for each bait, transfection conditions were selected to achieve equal expression levels in both HCT116 (mtKRAS^{Hi}) and HKE3 (mtKRAS^{Lo}) cells. For SILAC labelling we adopted protocols previously used for triple labeling [17-19]. Briefly, cells were grown in SILAC DMEM supplemented with 3mM L-Glutamine, 10% dialyzed FCS, 0.55mM lysine and 0.4mM arginine. For "Light" labelling L-[$^{12}C_6$,$^{14}N_2$]-lysine (Lys0) and L-[$^{12}C_6$,$^{14}N_4$]-arginine (Arg0) were added to the SILAC DMEM, for "Medium" labelling, L-[$^{2}H_4$]-lysine (Lys4) and L-[$^{13}C_6$]-arginine (Arg6), and for "Heavy" labelling, L-[$^{13}C_6$,$^{15}N_2$]-lysine (Lys8) and L-[$^{13}C_6$,$^{15}N_4$]-arginine (Arg10) were used. 0.5mM proline was added to all SILAC media to prevent arginine to proline conversion. All amino acids were purchased from Silantes. As we observed changes in expression for some baits caused by growth in SILAC media and in order to improve the accuracy of quantitation, we performed the AP-MS experiments using label swapping to correct for such expression changes. In line with our own experience, SILAC label swapping has been shown to reduce a major source of quantification error and to vastly improve the accuracy of quantitation [20]. In addition, label swapping requires twice as many replicates which improves data quality, and preys must be consistently identified in both labelling conditions providing an experimental design that reduces the number of false positive interactions. Cells were cultured in 150mm dishes in biological triplicate for each condition (Light, Medium, Heavy; n=3 forward and n=3 reverse samples per bait per cell line), according to the following scheme:

FORWARD	HCT116 Heavy, (Bait)	HKE3, Medium (Empty Vector)	HKE3 Light, (Bait)
REVERSE	HCT116 Light, (Bait)	HCT116, Medium (Empty Vector)	HKE3 Heavy, (Bait)

Twenty-four hours post transfection, cells were serum deprived for a minimum of 12 hours before cells were washed with phosphate-buffered saline (PBS; Thermo Fisher) and lysed with lysis buffer (1% NP40, 20mM Tris-HCl pH 7.5, 150mM NaCl, 1mM $MgCl_2$) supplemented with a protease inhibitor cocktail (Roche) and phosphatase inhibitors (2mM sodium orthovanadate, 10mM sodium fluoride and 10mM β-glycerophosphate; all from Sigma-Aldrich) for 10 minutes at 4°C. Lysates were cleared by centrifugation at 4°C and 20,000xg for 10 minutes. Total protein concentration in supernatants was determined by the Pierce BCA assay following the manufacturer's instruction. Equal concentrations of total protein lysates were incubated with 10µl of FLAG-M2 agarose beads (Sigma-Aldrich) for 2 hours at 4°C under constant agitation. The beads were washed twice with lysis buffer, and corresponding SILAC samples were combined. Then, beads were washed 3x with TBS (10mM Tris-HCl, 150mM

NaCl) to remove detergent.

10. Preparation of immunoprecipitates for mass spectrometry analysis. Following the wash steps, the proteins were either digested on-beads [21] or eluted by addition of 200µl FLAG peptide (200µg/ml) in TBS followed by methanol-chloroform precipitation and in-solution digest as described earlier [22]. Trypsin digestion was carried out in 2M Urea, 50mM Tris-HCl pH 7.5, 1mM DTT containing 5µg/ml modified sequencing-grade trypsin (Promega) overnight at 28°C. Peptides were alkylated using iodoacetamide (5mg/ml) and incubated in the dark for 30 minutes at room temperature. Samples were desalted using C18 Stage Tips as described [23] and analyzed by mass spectrometry.

11. Mass spectrometry (MS) analysis of immunoprecipitates (affinity purification-MS, AP-MS) was performed on Orbitrap instruments. In-solution digests were analyzed on an LTQ Orbitrap Velos mass spectrometer coupled to an Ultimate3000 RSLC system (Thermo Scientific). Tryptic peptides were automatically loaded onto a nanotrap column (75µm i.d. × 2cm, packed with Acclaim PepMap100 C18, 3µm, 100Å; Dionex) at a flow rate of 6µl/minute in 98% buffer C (0.1% trifluoroacetic acid in HPLC-grade water) and 2% buffer B (80% acetonitrile and 0.08% formic acid in HPLC-grade water). After 3 minutes, peptides were eluted and separated on the analytical column (75µm i.d. × 25 cm, Acclaim PepMap RSLC C18, 2µm, 100Å; Dionex) by a linear gradient from 2% to 30% of buffer B in buffer A (2% acetonitrile and 0.1% formic acid in HPLC-grade water) at a flow rate of 300nl/min over 85 minutes. Remaining peptides were eluted by a short gradient from 30% to 95% buffer B over 10 minutes. The eluted peptides were injected online into the MS. From the MS1 scan (resolution: 30 000, two miroscans per spectrum) with a mass range of 300–1,500 the 10 most intense peptide ions were selected for fragment analysis in the linear ion trap if they exceeded an intensity of at least 200 counts and if they were at least doubly charged. The normalized collision energy for collision-induced dissociation was set to a value of 35, and the resulting fragments were detected with normal resolution in the linear ion trap. Every ion selected for fragmentation was excluded from re-selection for 20 seconds by dynamic exclusion. On-bead tryptic digests were analyzed on an Ultimate Ultra 3000 chromatography system coupled to a Q-Exactive mass spectrometer (Thermo Fisher Scientific). Tryptic peptides were automatically injected on a homemade column (100mm length, 75mm inside diameter) packed with 1.8µm Reprosil AQC18 (Dr Maisch), using an increasing linear acetonitrile gradient from 2 to 40% of buffer B in buffer A (2% acetonitrile and 0.1% formic acid in HPLC-grade water) at a flow rate of 250nl/minute over 40 minutes. Full scan resolution was set to 70,000 and mass range was set to 350-1,600 selecting the 12 most intense ions for MS/MS. The lock mass option was activated for calibration using a background mass of 445.12003 [24].

12. Analysis of AP-MS data. The resulting mass spectra were analyzed using the MaxQuant software suite [25] (v 1.3.0.5) containing the in-built Andromeda search engine to identify the proteins from the UniProt HUMAN database (release 2014_02) containing 20,242 entries. The following MaxQuant parameters were used: trypsin was selected as enzyme, with two missed cleavages per peptide allowed; fixed amino acids modification was carboamidomethylation of cysteines; variable amino acids modifications were oxidation in methionine and acetylation in protein N-terminus; first search 20ppm and main search 6ppm with fragment ion mass tolerance set to 0.5 Da; 0.01 False Discovery Rate for analyses at both peptide and protein levels. To perform protein quantification the following criteria needed to be met: a minimum of one unique peptide with a minimum length of six amino acids, and a minimum ratio count of two. Contaminants were automatically excluded by enabling the MaxQuant contaminant

database search. In summary, we performed 1,710 immuno-purifications and 1,140 MS analyses consisting of 3 biological and 2 technical replicates with forward and reverse SILAC labelling of 95 baits and empty vector controls in 2 cell lines. The discrepancy between sample numbers and the number of MS analyses originates from the fact that SILAC triplets (light, medium, heavy) were pooled before MS analyses, and because the MS analyses include 2 technical replicates for each sample (i.e. the same sample was analyzed twice by MS).

In order to address the issue of eliminating false positive interactions without compromising the retention of true positive interactions, we had to consider that (i) our data are not from a single cell system but a comparison across two different cell lines where binders may be differentially expressed; and (ii) that we started from a biased bait selection. Our baits were chosen to represent key nodes of the EGFR signaling network based on prior knowledge. Thus, we can expect many shared preys between baits (as there are). These conditions, i.e. potential differences in prey expression between cell lines and many shared preys due to biased bait selection, prohibited the use of popular AP-MS analysis packages, such as SAINT [26]or CompPASS [27] that take co-precipitating proteins across many samples as unspecific background binders. In order to avoid these potentially confounding issues we used HiQuant, a software that we have previously developed [28]. As the first analysis step, significant prey proteins were identified in comparison to empty vector controls. Each prey protein was quantified in each pull-down experiment using 12 replicates (n=3 biological replicates * 2 technical replicates * forward and reverse labelling) in comparison to empty vector control replicates (also n=12). After removing contaminant proteins, normalizing the data and adjusting for missing data, a Student's t-test was computed to assess whether the prey protein abundance in the pull-down replicates was significantly greater than the empty vector control replicates. Next, prey proteins which were statistically significant in the t-test ($P<0.05$) were further assessed. For a given bait, the log2(bait/empty vector control background) of all significant prey proteins were assessed using the significance A statistical test, a Q-function tail probability test that is widely used in proteomics [25]. Essentially, this test selects for prey proteins that are at the extreme tail of the distribution (95%ile) of all t-test significant prey proteins for a given bait protein. Only prey proteins which also have significance A < 0.05 (and pass all the other criteria) were selected as likely true positive interactors. This excludes the vast majority of interactors that were identified vs empty vector controls alone. Additionally, to assess an interaction as statistically rewired it had to be both significant in the comparison to empty vector controls (as outlined above) and must also have been statistically significantly different from the same bait-prey interaction in the other cell line (also assessed using the significance A test but comparing the data from the 2 different cell lines). Data with fewer than 3 replicates were discarded. Compared to currently available methods, HiQuant resulted in an estimated 200-fold improvement in execution time for the post-quantification data analysis.

To benchmark the stringency of our analysis pipeline, we analysed 5 highly rewired baits with SAINTexpress, a commonly used AP-MS software that uses common binders as reference to eliminate false positive interactions. The results show that our analysis is much more stringent (Supplementary Table 4).

	**No of prey identified with our analysis**		**No of prey identified with SAINT**	
**Bait**	**HCT11**	**HKE3**	**HCT116**	**HKE3**
GRB2	74	52	169	156
PDPK1	59	27	148	165
PRKD1	55	74	362	366
PTK6	24	39	102	97
SH2D3C	81	74	526	521

Supplementary Table 4. *Results of 5 highly rewired baits using SAINTexpress and a SAINTscore BFDR <0.05 (indicating highly valid interactions) as a cut-off.*

Furthermore, as the networks were constructed from independent pull-down experiments in 2 different cell lines we could assess a minimum threshold for true positive interactions. At this threshold, 70% of the interactions were in common between the two cell lines indicating that the majority of interactions are true interactions (i.e. 70% at least and that assumes there are no real differences between cell lines which is clearly not the case). Additionally, comparison to several other publicly available AP-MS datasets reveals a similar proportion of novel interactions detected by us as detected in other major AP-MS mapping studies (e.g. Bioplex2.0).

AP-MS data were deposited in the PRIDE database under the following accession numbers: PXD016512, PXD016505, PXD016465, PXD016464, PXD016463, PXD016462, PXD016461.

13. Sample preparation for protein expression profiling by MS. HCT116 ($mtKRAS^{Hi}$) and HKE3 ($mtKRAS^{Lo}$) cells were grown in either "light" or "heavy" SILAC DMEM medium as shown below with 3 biological replicates for each labelling condition:

FORWARD	HCT116 Heavy	HKE3 Light
REVERSE	HCT116 Light	HKE3 Heavy

When the cells reached 70-80% confluency, they were serum starved for 12 hours and lysed in 1% NP40, 20mM Tris-HCl pH 7.5, 150mM NaCl, 1mM $MgCl_2$ supplemented with protease inhibitor tablets (Roche) and phosphatase inhibitors (2mM sodium orthovanadate, 10mM sodium fluoride and 10mM β-glycerophosphate, all Sigma-Aldrich), for 10 minutes at 4°C. Lysates were cleared of debris by centrifugation at 20,000xg at 4°C for 10 minutes. Total protein concentration in supernatants was measured by the Pierce BCA assay as per manufacturer's instruction. Equal amounts of protein from each cell line were mixed 1:1 and loaded on 10% precast Tris-Glycine SDS-polyacrylamide gels (Thermo Fisher Scientific). The gel was cut in 10 slices, and subjected to in-gel tryptic cleavage as previously described [29].

14. MS analysis of protein expression profiles was performed on an Ultimate3000 RSLC system coupled to an Orbitrap Fusion Tribrid mass spectrometer (Thermo Fisher Scientific). Tryptic peptides were loaded onto a nano-trap column (300µm i.d. × 5mm precolumn, packed with Acclaim PepMap100 C18, 5µm, 100Å; Thermo Scientific) at a flow rate of 30 µl/min in 0.1% trifluoroacetic acid in HPLC grade water. After 3 minutes, peptides were eluted and separated on the analytical column (75µm i.d. × 25cm, Acclaim PepMap RSLC C18, 2µm, 100Å; Thermo Fisher Scientific) by a linear gradient from 2% to 30% of buffer B (80% acetonitrile and 0.08% formic acid in HPLC-grade water) in buffer A (2% acetonitrile and 0.1% formic acid in HPLC-grade water) at a flow rate of 300nl/minute over 150 minutes. Remaining peptides were eluted by a short gradient from 30% to 95% buffer B in 10 minutes. MS parameters were as follows: for full MS spectra, the scan range was 335–1,500 with a resolution of 120,000 at m/z=200. MS/MS acquisition was performed in top speed mode with 3 seconds cycle time. The maximum injection time was 50ms. The AGC target was set to 400,000, and the isolation window was 1 m/z. Positive Ions with charge states 2-7 were sequentially fragmented by higher energy collisional dissociation. The dynamic exclusion duration was set to 60 seconds and the lock mass option was activated and set to a background signal with a mass of 445.12002 [24].

15. Analysis of protein expression profiling MS data was performed using the MaxQuant software (version 1.5.3.30). Trypsin was selected as the digesting enzyme with maximal 2 missed cleavages. Cysteine carbamidomethylation was set for fixed modifications and oxidation of methionine and N-terminal acetylation were specified as variable modifications. The data were analyzed with the minimum ratio count of 2. The first search peptide tolerance was set to 20, the main search peptide tolerance to 5ppm and the "re-quantify" option was selected. For peptide and protein identification the Human subset of the SwissProt database (Release 2015_12) was used, and contaminants were detected using the MaxQuant contaminant search. A minimum peptide number of 1 and a minimum length of 6 amino acids was tolerated. Unique and razor peptides were used for quantification. The match between run option was enabled with a match time window of 0.7 min and an alignment time window of 20 min. The statistical analysis including ratio, two sample *t*-test and significance A calculation was done using the Perseus [30] and the HiQuant softwares [28]. The protein expression profiling data were deposited in the PRIDE database under accession number PXD016549.

16. Sample preparation for MS based phosphoproteomics. Serum starved HCT116 (mtKRAS^{Hi}) and HKE3 (mtKRAS^{Lo}) were lysed in 8M urea and protein concentration was determined using a BCA protein assay (Bradford Biorad). 500 µg of protein extracts were digested with 5µg/ml trypsin in 2M urea, 25mM ammonium bicarbonate, and 5mM dithiothreitol at room temperature overnight. Cysteines were alkylated using 10mM iodoacetamide for 1 hour. Tryptic peptides were purified on an SPE reverse phase Bond Elut LMS cartridge, 25mg (Agilent), dried under low pressure and stored at -20°C. Then, phosphopeptides were enriched using TiO_2 beads. Briefly, 25µl of 0.5M lactic acid in 50% acetonitrile was added to the samples. After sonication, the sample was added to 8µl resin (100µg/µl of 10µm TiO_2 beads in isopropanol) and incubated overnight. Then, the sample/TiO_2 mixture was transferred to a filter and spun for 5 minutes. The resin on the filter was washed twice with 25µl of 0.5M lactic acid in 50% acetonitrile, and once with 200µl 80% acetonitrile in 0.1% TFA. Phosphopeptides were eluted twice with 50µl of 50mM KH_2PO_4 followed by further elutions with 50µl of 2M ammonia and 50µl of 80% acetonitrile in 0.1% TFA. Eluates were dried in a speedvac and then reconstituted in 100µl Buffer A for desalting in C18 stage tips. Peptides were dried under low pressure, reconstituted in 7µl 0.5%v/v trifluoroacetic acid to a final concentration of 1µg/µl and filtered through a 0.45µm filter.

17. MS analysis of phosphopeptides was performed using an Ultimate3000 RSLC system coupled to a Q-Exactive instrument (Thermo Fisher). The pre-column of 300µm x 5mm (Acclaim Pepmap, 5µm particle size) was connected to a column of 75µm x 50cm (Acclaim Pepmap, 3µm particle size). Peptides were separated using a 90 minutes gradient under the following conditions: 7 minutes with buffer A (2% acetonitrile, 0.1%formic acid), over 1 minute increase to 4% buffer B (80% acetonitrile, 0.1% formic acid), over 57 minutes increase to 25% buffer B, over 4 minutes increase to 35% buffer B, over 1 minute increase to 98% buffer B for 9 minutes, then switching to 2% buffer B over 1 minute. MS analysis used data acquisition with a survey scan at 70,000 resolution followed by selecting the top 10 peptides for fragmentation and MS/MS analysis. Data were acquired using Xcalibur version 3.1.66.10.

18. Phosphoproteomics data analysis. MS/MS spectra were searched using MASCOT Version 2.4 (Matrix Science Ltd) against the human subset of the UniProt database with the maximum missed-cut value set to 2. The following features were used in all searches: (i) variable methionine oxidation, (ii) fixed cysteine carbamidomethylation, (iii) precursor mass tolerance of 10ppm, (iv) MS/MS tolerance of 0.05amu, (v) significance threshold (p) below 0.05

(MudPIT scoring), and (vi) final peptide score of 20. Progenesis version 4 (Nonlinear Dynamics) was used for label-free quantitation. Only MS/MS ions with a charge of 2+, 3+ or 4+ were taken into account for the total number of 'Features' (signal at one particular retention time and m/z), and only the five most intense spectra per 'Feature' were included. Normalization was first performed based on the median of the ion intensities of these sets of multi-charged ions. Where the same peptide was detected multiple times, the one with the highest Mascot score was retained. The associated unique peptide ion intensity was transformed using an ArcSinH function (a log transform is not ideal considering the significant amount of near zero measurements generated by the current method of detection). For the quantitative analysis we used the intensities of the representative peptides having the highest Mascot scores. Based on the abundance values, within group means were calculated and from there the fold changes (in comparison to control) were generated. One-way ANOVA testing was used to calculate the p-values based on the transformed values. Differentially phosphorylated proteins were only considered significant if the following conditions were fulfilled: (i) pairwise p-values < 0.05, (ii) number of peptides detected and used in quantification per protein equal to or more than 1 peptide, and (iii) absolute fold change at least 1.5 (i.e. ≥1.5 fold for up-regulated proteins or ≤ 0.667 fold for down-regulated proteins). The phosphoproteomics data were deposited in the PRIDE database under accession number PXD016431.

<u>19. Construction of protein-protein interaction networks (PPIN) and protein abundance and phosphorylation enrichment analysis.</u> The EGFRNet$^{mtKRAS\text{-}Hi}$ and EGFRNet$^{mtKRAS\text{-}Lo}$ networks were separately constructed by combining bait-prey interactions from each of the 95 chosen baits. Bait-prey interactions were included in the networks, if the abundance of the prey protein in the pull-down was significantly higher ($P \leq 0.05$) than in empty vector controls and the significance A value for the prey protein was also ≤ 0.05. To identify interactions that were significantly "rewired" in the HCT116 (EGFRNet$^{mtKRAS\text{-}Hi}$) network compared to the HKE3 (EGFRNet$^{mtKRAS\text{-}Lo}$) network, we used HiQuant to directly compare the SILAC data from the two cell lines. We defined interactions as being "rewired" in EGFRNet$^{mtKRAS\text{-}Hi}$, if the abundance of the prey protein in the pull-down was significantly different ($P \leq 0.05$) compared to EGFRNet$^{mtKRAS\text{-}Lo}$ and the significance A value for the prey protein was also ≤ 0.05. Interactions that were identified in only one EGFRNet, but where prey abundance was subsequently not found to be statistically significantly different in the respective bait-prey complexes in the two cell lines were not considered as rewired interactions. The top rewired nodes in EGFRNet$^{mtKRAS\text{-}Hi}$ were identified as those with most rewired interactions. The topological properties of the networks including node degree, betweenness centrality, clustering coefficient and network scale-freeness were analyzed using the NetworkAnalyzer application [31] in Cytoscape 3 [32]. Cytoscape session files for the EGFRNet$^{mtKRAS\text{-}Hi}$, EGFRNet$^{mtKRAS\text{-}Lo}$ networks can be provided upon request.

To investigate whether nodes in the EGFRNet$^{mtKRAS\text{-}Hi}$ network were enriched for differentially abundant proteins, a hypergeometric test was performed with the following parameters:

$$p(X \geq k) = \sum_{x=k}^{n} \frac{\binom{K}{x}\binom{N-K}{n-x}}{\binom{N}{n}},$$

N = total number of proteins assayed in the protein expression analysis.
n = total number of differentially abundant proteins identified.
K = number of proteins in the EGFRNet$^{mtKRAS\text{-}Hi}$ network that were assayed in the protein expression analysis.
k = number of differentially abundant proteins observed in the EGFRNet$^{mtKRAS\text{-}Hi}$ network.

A similar analysis was conducted to determine whether rewired nodes were enriched for differentially abundant or phosphorylated proteins.

20. Known protein complexes in the $EGFRNet^{mtKRAS-Hi}$ and $EGFRNet^{mtKRAS-Lo}$ networks. To identify high-confidence protein complexes in the EGFRNet PPINs, we integrated our network data with data from CORUM, a curated database of experimentally determined mammalian protein complexes [33]. The May 2017 release features 2,390 human complexes. CORUM complexes, where at least 70% of the component proteins in the complex were identified as nodes in an EGFRNet, were defined as being "present" in that network. Complexes were independently identified in the two EGFRNets.

21. Comparisons of the $EGFRNet^{mtKRAS-Hi}$ and $EGFRNet^{mtKRAS-Lo}$ networks to other publicly available datasets. To evaluate the EGFRNets in comparison to publicly available interaction data we compared them to four different datasets: (i) the biophysical interactions of ORFeome-based complexes (BioPlex 2.0) dataset constructed using high-throughput AP-MS, which identified the interacting partners of 5,115 bait proteins in HEK293T cells featuring 56,553 interactions [34]. 34 of the bait proteins in BioPlex 2.0 were also bait proteins in our experiments; (ii) a yeast two-hybrid (Y2H) dataset consisting of 13,017 binary interactions among 4,144 proteins [35]. 32 proteins in this dataset were baits in our experiments; (iii) a dataset of interactions annotated in the Integrated Interaction Database (IID version 2018-11), which integrates data from major publicly available interaction databases and contains 334,315 experimentally detected interactions among 17,776 proteins [3,4] and (iv) a dataset of 28,500 interactions identified from HeLa cells using a quantitative proteomics workflow to identify 28,500 interactions among 5,400 proteins [36].

554 interactions in $EGFRNet^{mtKRAS-Hi}$ (~18%) and 538 in $EGFRNet^{mtKRAS-Lo}$ (~19%) were annotated as experimentally validated human interactions in IID suggesting that >80% of the interactions detected in our current study are novel interactions. This fraction of newly discovered interatcions is comparable to other large-scale affinity purification mass spectrometry (AP-MS) studies such as BioPlex 2.0 [34], which reported that 87% of the interactions detected in HEK293 cells were novel. 34 of the bait proteins in our study were also baits in the BioPlex 2.0 dataset, and 30% of the interactions detected for these baits in BioPlex 2.0 were also identified in our study. Additionally, 18 baits in our study were also baits in another large-scale AP-MS study in HeLa cells [36], and ~13% of these bait-prey interactions were also detected in our study (which is comparable to the overlap between Hein et al. and BioPlex 2.0). Comparing interactions in $EGFRNet^{mtKRAS-Hi}$ and $EGFRNet^{mtKRAS-Lo}$ to a large-scale yeast two-hybrid (Y2H) dataset [35] we found little overlap (15 interactions), highlighting the well-known complementary nature of AP-MS and Y2H experiments.

Our study identified an average of ~30 interactions per bait. Comparing this number to interactions identified in other large-scale PPI studies suggests that our results are within a very plausible range. The global PPI Bioplex study identified an average of 10 interactors per bait [34]. Another global PPI screen found an average of 25.5 interacting proteins per bait [36]. While these global studies report a relatively small number of interactions per bait protein, studies focusing on a smaller number of baits in order to map pathways, report more PPIs even under stringent exclusion conditions. For instance, a PPI study of the centrosome-cilium generated >7,000 interactions using 58 bait proteins, i.e. >120 interactions per bait [37]. Similarly, an analysis of human host cellular proteins binding to Zika virus proteins revealed an average of 122 binding partners per bait [38]. The latter three studies used SAINT [26] for the analysis of AP-MS data. Such PPI numbers do not seem to be exaggerations observed in cultured cell lines, as studies in primary cells identified 53 high confidence interactors of the Grb2 protein [39]. These

comparisons strongly indicate that our analysis does not over-report PPIs.

<u>22. Equilibrium binding of RAS binding partners to RAS-GTP.</u> RAS effectors bind to a single site on RAS, and hence multiple binding partners compete for binding to the active RAS conformation. In order to determine how the concentrations of KRAS-effector complexes change with the concentration of active RAS in mtKRAS^{Hi} and mtKRAS^{Lo} cells, we developed a dynamic mathematical model. The competition is described by the following equilibria

$$RX_i = \frac{R * X_i}{K_i}, \qquad i = 1, \dots, N \tag{1}$$

Here X_i and R are the concentrations of free partner and free RAS-GTP, RX_i is the complex concentration, K_i is the dissociation constant (K_d) of the X_i binding (Supplementary Table 5).

Binding partner	K_d value	Reference
RAF1	0.08 µM	[40]
RALGDS	1.3 µM	[41]
RASSF5	0.4 µM	[42]
AFDN	3.0 µM	[43]
RIN1	0.022 µM	[44]
TIAM	1 µM	No data, estimated
PLCε	0.82 µM	[45]
PI3K	204.7 µM	[40]

***Supplementary Table S5**. K_d values of RAS bunding partners.*

We denote by aR^{Tot} the total active RAS concentration (i.e., RAS-GTP) and by X_i^{Tot} the total partner X_i abundance. The following equations describe the balance of all forms (moiety conservation):

$$R + \sum_{i=1}^{N} \frac{R * X_i}{K_i} = aR^{Tot} \tag{2}$$

$$X_i + \frac{R * X_i}{K_i} = X_i^{Tot} \tag{3}$$

From Eq. 3, we can express X_i in terms of R, X_i^{Tot} and K_i, as follows,

$$X_i = \frac{X_i^{Tot}}{1 + R/K_i} \tag{4}$$

Substituting Eq. 4 into Eq. 2, we readily obtain the free active RTK concentration, R, from the following equation,

$$R\left(1 + \sum_{i=1}^{N} \frac{X_i^{Tot}}{K_i + \mathrm{R}}\right) = aR^{Tot} \tag{5}$$

Each free concentration X_i is obtained by substituting the solution R of Eq. 5 into Eq. 4 and each complex concentration RX_i is obtained from Eq. 1.

23. A simplified solution of the equilibrium binding model. We partitioned RAS-GTP binding partners according to their K_d's (denoted by K_i, Eq. 1) into two groups. The low affinity group included partners with K_i that are larger than R^{Tot} ($K_i \gg aR^{Tot}$) and high affinity group comprised partners with K_i that are smaller than R^{Tot} ($K_i \leq aR^{Tot}$). If $K_i \gg aR^{tot}$, Eq. 1 can be approximated as a linear function of the free RAS-GTP concentration,

$$X_i^{Bound} \approx \frac{R * X_i^{Tot}}{K_i} \quad (6).$$

Eq. 6 means that the RAS-bound fractions of partners with large $K_i \gg aR^{tot}$ are proportional to their total abundances divided by K_i. Accordingly, the concentrations of free partners was approximated as,

$$X_i \approx X_i^{Tot}(1 - R/K_i) \quad (7).$$

Eq. 7 is the linear term of the Taylor expansion of Eq. 4. Using this approximation, the balance Eq. 5 can be written as follows, where large K_i correspond to $K_i \gg aR^{tot}$ and small K_i correspond to $K_i \leq aR^{Tot}$

$$R\left(1 + \sum_{i,\ Large\ Ki} \frac{X_i^{Tot}}{K_i} + \sum_{i,\ small\ Ki} \frac{X_i^{Tot}}{K_i + \mathrm{R}}\right) \approx aR^{Tot} \quad (8).$$

If $K_i \ll aR^{Tot}$, we can conclude from the above equations that the RAS-bound concentrations for such partners will be determined by the total abundances, X_i^{Tot}, in a sharp contrast to partners with the large K_d's ($K_i \gg aR^{tot}$). The bound fractions of proteins that bind with K_i less than aR^{tot} but greater than very small K_i can be found by solving Eq. 8. Numerical calculations showed that the bound levels of these proteins mainly depend on the total abundances X_i^{Tot} (although they also slightly depend on K_i and aR^{tot}).

24. Changes in the abundances of KRAS-effector complexes with KRAS-GTP concentrations. Signaling by KRAS-effector complexes (about 10 putative KRAS effectors) initiates downstream KRAS effector pathways dependent on the formation of specific KRAS effector complexes. When the active KRAS concentration is small (≤ 100 nM), high-affinity partners ($K_i \leq 10 - 100$ nM) dominate KRAS-effector complexes. As active KRAS increases, low-affinity partners bind to RAS, while tightly-bound fractions of very high affinity binders hardly change (see Eqs. 1-8). Because the abundance of some low-affinity partner (e.g., RALGDS) is much greater than the high-affinity partner abundance (such as RAF1), at certain KRAS-GTP levels, the bound concentration of a low-affinity partner starts to exceed the bound concentration of a high-affinity partner (compare concentrations of RAF1-KRAS and RALGDS-KRAS complexes in Fig. 5A of the main text). Likewise, the relative fractions of KRAS-high and low affinity effector complexes dramatically depend on the mutant KRAS dosage, because a group to which a particular partner belongs changes with the KRAS-GTP abundance. The model allows us to rank KRAS binding partners according to the fold-changes in KRAS-bound fractions with the change in mtKRAS. The fold-changes equal RX_i^{hi}/RX_i^{lo} ratios (Eq. 1), where RX_i^{hi} and RX_i^{lo} are the concentrations of ith partner complex with KRAS for high and low doses of mtKRAS, respectively. In the model KRAS-GTP was changed from 70 nM (low mtKRAS) to 300 nM (high mtKRAS). Importantly, the ranking of KRAS binding

partners by their fold-changes in KRAS-bound fractions did not change with simultaneous changing of low and high KRAS-GTP levels by 100%.

25. EGFR network rewiring caused by the change in RAS dose for HCT116 and HKE3 cells. Our interaction proteomics data show fold-changes of protein-protein complexes that were measured for high and low mtKRAS doses in HCT116 and HKE3 cells. We selected baits that belong to known RAS effector pathways. The baits were components of the RAF/MAPK, RAL, PI3K, TIAM, AFDN, PLCε, and RIN pathways. For each bait (i) belonging to RAS effector pathway (k) we considered statistically significant fold-changes (F_{ij}^{k}) of its protein-protein complex with prey (j), measured for high and low mtKRAS,

$$F_{ij}^{k} = \frac{I_{ij}^{k\,hi}}{I_{ij}^{k\,lo}}, \qquad j = 1 \ldots N_i^k, i = 1 \ldots M_k \tag{9}$$

Here N_i^k is number of preys detected for bait i, $I_j^{k\,hi}$ and $I_j^{k\,lo}$ are the measured concentration of the bait i - prey j complex in arbitrary units in cells with high and low levels of mtKRAS, respectively. Next, for each bait (i) - a node of a particular KRAS effector pathway (k), we averaged the logarithms of fold-changes in the complexes, which contained this bait and were statistically significant

$$A_i^k = \frac{1}{N_i^k} \cdot \sum_{j=1}^{N_i^k} \log F_{ij}^k \tag{10}$$

These values (A_i^k) characterize the sensitivity of the nodes to the alteration in mtKRAS dose. Then, we calculated the sum of the absolute values of the sensitivities of nodes that belong to each KRAS effector pathway (normalized by the number of pathway nodes whose sensitivities were measured),

$$S_k = \frac{1}{M_k} \cdot \sum_{i=1}^{M_k} |A_i^k| \tag{11}$$

The normalized sum S_k can serve as the experimentally measured metric of the overall change in the KRAS effector pathway signaling in response to two different doses of KRAS-GTP. It is also a metric of the pathway rewiring due to change in the mutant KRAS dose. We then ranked these KRAS effector pathways accordingly to this metric and compared the model-predicted ranks of KRAS-effector complexes (that initiated KRAS-effector pathways) with the ranks of the KRAS effector pathways based on the interaction proteomics data (Supplementary Data 13).

26. Information Flow Analysis of EGFRNet$^{\text{mtKRAS-Hi}}$ and EGFRNet$^{\text{mtKRAS-Lo}}$ networks. We considered a number of options for analyzing how the EGFR PPI network transduces information. These included more abstracted models, such as Boolean networks, Bayesian models, and data driven models to detailed mechanistic models, such as ODE models [46-49]. All these methods have their advantages and limitations. Major limitations include the large size of our networks (>3000 PPIs in each network) and the type and amount of data required, which rule out the use of mechanistic ODE models. Given these constraints and the aim to explore the functional effects of topological network changes on information flux, we employed a computational modelling approach called information flow (IF) analysis [50,51]. This approach has a number of advantages. It can be applied to large, undirected networks such as the one in

this study. It is computationally efficient and allows competing hypotheses to be investigated (e.g. comparing flow in 2 different networks).

To perform IF analysis from the EGFR at the cell membrane to nuclear transcription factors (TFs), the two EGFRNets were first supplemented with publicly available prey-prey interactions. Additionally, the networks were also supplemented with 122 additional nodes that are known to be involved in EGFR signaling [14] but were not chosen as bait proteins in our AP-MS experiments (Supplementary Data 14). Prey proteins and the additional nodes were uploaded to InnateDB.com [52] to retrieve all the publicly available interactions for these nodes. These networks are referred to as the HCT116^{IFANET} and HKE3HKE3^{IFANET} and are available in JSON format upon request. The HCT116^{IFANET} consisted of 1,443 nodes and 5,633 edges and the HKE3HKE3^{IFANET} consisted of 1,345 nodes and 5,440 edges. Information flow analysis was implemented using the CytoITMprobe software (damping factor = 0.85; channel model selected) [53]. Information flow analysis was performed separately on the HCT116^{IFANET} and HKe-3^{IFANET} networks, selecting EGFR as the source node of signaling and 19 downstream TFs (Supplementary Data 14) as the sinks for the information flow. The more signals that are simulated to flow through a node, the higher the information flow score of that node will be. Information flow scores for each node in both the HCT116^{IFANET} and HKE3HKE3^{IFANET} networks were determined.

27. Gene Ontology, pathway and transcription factor binding site analyses. Gene Ontology (GO) and pathway analyses were performed using InnateDB.com [52]. GO terms or pathways that had an FDR < 0.05 were identified as significantly enriched. Transcription factor binding site analysis was undertaken using the findMotifs.pl program in HOMER v4.8 [54], with the human hg38 promoter set in order to identify enriched motifs.

28. Development of the PRIMESDB platform. PRIMESDB is an online web resource designed to facilitate exploration of the AP-MS PPI data generated in this study (which we call the PRIMES project) by the wider research community. It is accessible at *primesdb.eu*. PRIMESDB is an observer member of The International Molecular Exchange (IMEx) consortium, the international standards body for the curation and exchange of published protein-protein interaction data [55]. All PPI data generated in this study also been deposited with IMEx (IMEx accession number IM-26434). To explore each of the bait-prey protein complexes identified in either EGFRNet$^{mtKRAS\text{-}Hi}$ or EGFRNet$^{mtKRAS\text{-}Lo}$ users can click on the “PRIMES PPI DATA” tab on the PRIMESDB homepage. Select a bait protein of interest from the dropdown menu and click “Visualise with Cytoscape”. This will open a new page displaying the bait-prey interactions for the selected bait in the HCT116 (mtKRAS^{Hi}) and HKE3 (mtKRAS^{Lo}) cells or in a network displaying the union of all interactions detected in either cell line. In addition to the PRIMES PPI data, PRIMESDB also collates publicly available PPI data on the entire human and mouse interactomes from public repositories including the major IMEx repositories [55]. This information is stored in a purpose-built SQL database designed to allow efficient searching of PRIMESDB data from the web interface. The database also integrates PPI data with an extensive range of supplementary orthogonal data including KEGG [56] and Reactome [57] pathway annotations, Gene Ontology annotations [58], colorectal cancer mutation data from the Catalogue Of Somatic Mutations In Cancer (COSMIC) database [59], protein structure data from the RSCB protein data bank [60], drug target data from DrugBank [61], parent gene information from Ensembl [62], and protein expression data from the NCI60 panel [63] and from another dataset of 11 common cell lines for which the complete proteomic analysis has been performed [64]. Through PRIMESDB users can explore these data using several different search options. For example, users can upload a list of proteins and return the interactions that

those proteins are annotated to participate in. To facilitate visualization of this network data we have developed a web-embeddable network visualization tool called CerebralWeb, which lays out molecular interaction networks based on the subcellular locations of the nodes [65]. Dynamic interaction data, such as that generated in this study, present a special challenge in network biology. To overcome these challenges and as part of this study, we have also developed DyNet, a Cytoscape application that provides a range of functionalities for the visualization, real-time synchronization, and analysis of large multi-state dynamic molecular interaction networks enabling users to quickly identify and analyze the most 'rewired' nodes across many network states [66]. PRIMESDB also has a pathway analysis tool to enable users to identify statistically enriched pathways in an uploaded list of proteins of interest.

29. Analysis of CRC patient data. We obtained the survival data of 629 CRC patients from the TCGA dataset [67] (https://www.cbioportal.org/study/summary?id=coadread_tcga), and correlated alterations in genes encoding either the top 20 most rewired or the bottom 20 least rewired bait proteins (as defined by the number of rewired interactions) with survival. The alterations included were mutations, copy number changes, mRNA expression changes, and protein expression changes. As an additional control we also selected a set of 36 baits that accounted for the same number of interactions as the top 20 baits in the network. The results were displayed using Kaplan-Meier curves [68]. The Kaplan-Meier curves were plotted using PRISM 7.0.3. To further assess the accuracy of the top-20 bait proteins to classify patients into high and low risk groups, we trained a Lasso classifier [69] using RNAseq expression, copy-number, and mutation data for the patients from TCGA. Lasso works by selecting the most important coefficients that are predictive of the two risk groups. Five-fold cross-validation of the classifier by subsampling the patient data into training (80%) and testing (20%) datasets gave an accuracy of up to 0.79 (mean 0.70) and an area under the ROC curve (AUC) of 0.763 (Supplementary Figure 9). A similar classification using the bottom-20 rewired proteins gave a much lower mean accuracy of 0.4 and AUC of 0.522. The two ROC curves were compared using the roc.test function in the pROC package with method option "DeLong's test" [70]. The statistical performance was calculated with pROC and found to be highly significant (p value: 1.248e-15).

30. Membrane yeast two-hybrid assay (MYTH). Increasing evidence suggests that in addition to EGFR (ERBB1) the other family members ERBB2/3/4 play a role in CRC and may provide mechanisms of drug resistance [71]. Integral membrane protein interactions are difficult to capture in AP-MS experiments [72]. Therefore, we applied MYTH, a membrane yeast two-hybrid assay, which detects interactions *in situ* by reconstitution of a split ubiquitin probe [73], to identify binary protein interactors of the human ERBB2, ERBB3, and ERBB4 proteins. The cDNAs encoding these human ERBB proteins (devoid of their endogenous cleavable signal sequence) were fused to the signal sequence of the yeast α-mating pheromone precursor (MFα) at their N-termini, and to the C-terminal part of ubiquitin (Cub) followed by an artificial transcription factor (TF) at their C-termini. The resulting MFα-ERBB-Cub-TF chimeric proteins correctly localized to the plasma membrane and lacked self-activation. We have also tested the phosphorylation status of all 4 ERBB receptors by probing total yeast protein extracts using antibodies specific for their phosphorylated tyrosine residues finding that ERBB2 and ERBB4 are phosphorylated in yeast whereas ERBB3 is not. This phosphorylation of ERBB2 and ERBB4 receptors in yeast is due to their homodimerization, since the mutant versions of both receptors in which their tyrosine kinase domains were inactivated by introducing previously described mutations were not phosphorylated [74]. After these tests, we screened a human fetal brain cDNA library fused to the mutated N-terminus of Ubiquitin (NubG) [73] for proteins that can associate with ERBB2, ERBB3, and ERBB4 proteins. The MYTH protein interaction

network (Supplementary Data 3) was constructed using the MYTH data generated in this study, previous MYTH-derived data for the EGFR from [75], known and predicted interactions from Integrated Interactions Database, IID ver. 2018-11 [3,4], and visualized in NAViGaTOR 3 [5]. Gene Ontology [58] Molecular Function was used to color nodes, where the decision to assign the category was based on the highest overlap between GO entries for the object and all the sublists of GO entries for the category. MYTH interactions were annotated using IID ver 2018-11 [3]. We included IID evidence type (column G in Supplementary Data 3), tissue annotation (columns J-AH), disease annotation (columns AJ-BI). To assess possible tissue-specific and disease-prevalent interactions, we used the Mann-Whitney Rank Sum Test, considering summary counts from columns H and I, respectively. Clearly, more tissues are annotated for MYTH interaction, and more MYTH-IID interactions are annotated with disease (Supplementary Figure 4B).

This ERBB2-4 interactome contained 197 interactions, of which 181 were new. Interestingly, ERBB2 and ERBB3 cover most of these interactions, which may be related to the enhanced signaling capacity and biological aggressiveness of cancers expressing ERBB2/3 dimers [71]. 29 proteins interacted with more than one ERBB receptor. MYTH has also previously been applied to identify EGFR interactors [75], and we integrated these data and data from the Integrated Interactions Database [3] with the ERBB2-4 interactome to construct a complete interactome map of all four ERBB receptors, comprising 405 interactions (Supplementary Figure 4 and Supplementary Data 3). Proteins interacting with ERBB1-4 participate in a wide range of functions, and were highly enriched in the KEGG ERBB signaling pathway (-log10(P) > 20), suggesting that the majority of these interactions are true interactors. Thus, this interactome provides a resource for further investigations of proximal ERBB signaling, especially as according to Gene Ontology analysis, 97 preys (~25%) are membrane proteins.

31. Immunoprecipitation and Western blotting. Cells were lysed in in 1% NP40 lysis buffer described above**.** Lysates were cleared by centrifugation at 20,000xg for 10 minutes, and adjusted to equal protein concentrations using the Pierce BCA assay kit. Equal concentrations of lysates were added to 10µl Flag agarose M2 beads (Sigma) or 10µl of Protein G Sepharose 4 Fast Flow beads (Sigma) coupled to the respective antibodies. After incubation at 4°C on a vertical rotator for 1 hour, immunoprecipitates (IPs) were washed 1x with 1% NP40 lysis buffer and 3x with TBS. Then, IPs were separated by sodium-dodecylsulfate polyacrylamide gel electrophoresis (SDS-PAGE) and transferred to Polyvinylidene difluoride (PVDF) membranes. Blots were incubated with the respective antibodies and developed using Enhanced Chemiluminescence (ECL; Thermo Fisher) according to the manufacturer's instructions. Blots were quantified using the Image J software. For activity assays phospho- or activation specific antibody signals were normalized to the total abundance of the respective proteins.

32. RAS pulldown assays. Dh5α *E. coli* transformed with a GST-RAF-Ras Binding Domain (RBD) construct were grown overnight at 37°C in 20ml of LB medium containing ampicillin. This culture was added to 500ml of LB medium and grown until the OD^{600} reached 0.6-0.8, and GST-RBD expression was induced by adding Isopropyl β-D-1-thiogalactopyranoside at a final concentration of 1mM for 2 hours at 37°C. The bacteria were collected by centrifugation, resuspended in 10ml of PBS (Thermo Fisher) containing 1% NP-40 (Calbiochem) and Leupeptin (10ug/ml; Sigma Aldrich), and sonicated using a Syclon Ultrasonic Homogenizer at 8-10% power using repeated 6 seconds on – 1 second off cycles for a total of 5 minutes. Following centrifugation at 4,000 rpm for 30 minutes at 4°C, the supernatant was incubated with 500µl of Glutathione Sepharose 4b beads (GE Healthcare) on a rotor wheel at 4°C by for

3 hours. Subsequently, samples were centrifuged at 4,000 rpm for 1 minute and beads were resuspended in 1ml of PBS containing 1% NP40 and subjected to 5 wash cycles: 2x PBS containing 1% NP40, 2x PBS and a final wash in magnesium lysis buffer (MLB: 25mM Hepes pH 7.5, 150mM NaCl, 1% NP40, 10% Glycerol, 25mM NaF, 10mM $MgCl_2$, 1mM EDTA, 1mM Na vanadate, 10µg/ml Leupeptin). Then, beads were resuspended in MLB buffer and stored at 4°C with protease inhibitors. The concentration of the GST-RBD on the beads was determined by comparing 10µl of GST-RBD beads against a bovine serum albumin (BSA) dilution series on a SDS polyacrylamide gel followed by Coomassie staining to visualize the proteins. To determine the amount of active KRAS in the $mtKRAS^{Hi}$ $mtKRAS^{Lo}$ cell lines, cells were washed in PBS and lysed in PBS containing 1% NP40. Lysates adjusted to equal protein concentrations were incubated with the GST-RBD beads for 1 hour at 4°C. Beads were collected by centrifugation and washed 3x with MLB buffer. Active KRAS captured by the GST-RBD beads was detected by Western blotting with KRAS antibody (sc-30, F234; Santa Cruz).

33. Cell proliferation assays. 50,000 cells/well were seeded in 6 well plates and grown in DMEM with 10% FCS for 72 hours. Cells were washed twice with PBS and stained with 1ml 0.2% crystal violet (Sigma) in 20% methanol for 10 minutes. Excess staining solution was removed by 3 washes with H_2O, and plates were dried completely. The crystal violet retained by the cells was solubilized with 0.5 ml 10% acetic acid solution by gentle shaking for 20 minutes. Solubilized crystal violet was diluted 1:5 in H_2O and absorbance was measured at 590nm against 2% acetic acid solution as blank. Experiments were performed in triplicate.

34. Colony forming assays. 5,000 cells were seeded in 100mm dishes in DMEM supplemented with 10% FCS. Medium was replenished every 3 days. After 2 weeks or when colonies became microscopically visible, cells were washed twice with PBS and stained with 0.2% crystal violet (Sigma) in 20% methanol for 10 minutes. Cells were washed 3 times with H_2O to remove excess stain and dried completely before being imaged. ImageJ plugin ColonyArea [76] was used to quantify colonies. All experiments were carried out in triplicate.

35. Anchorage Independent Growth Assays were carried out as previously described [77]. Briefly, 6-well plates were coated with a layer of 0.6% agar (Sigma) in DMEM supplemented with 20% FBS. 10,000 cells were resuspended in 0.3% low melting point agar with DMEM containing 20% FBS at 42°C and immediately seeded onto the coated plates. After the agar had solidified, it was overlaid with 1 ml DMEM plus 20% FBS, which was changed twice a week. On day 20 media was discarded, and soft agar colonies were stained with 1ml Crystal Violet (0.01% in 10% EtOH) for 30 minutes at room temperature. Plates were then washed 3x with H_2O for 30 minutes each. The fourth wash was left for 5 hours to remove any background staining. Plates were imaged on an Epson Perfection V750 Pro Scanner, and colonies were quantified using the ColonyArea plugin for ImageJ [76]. Experiments were performed in triplicate.

36. Cell migration was measured in a 96-well plate using the Oris™ 2D Cell Migration Assay (Platypus technologies) as per the manufacturer's protocol. Briefly, silicone stoppers were added to the wells forming a central circle area of 2mm. $3x10^4$ cells were seeded per well and let adhere overnight. The next day stoppers were removed, and wells were visually inspected to ensure the central area was cell free. "No migration" control stoppers were left until the end of the experiment. After 48 hours, the migration into the central void was analyzed by fluorescence microscopy after staining cells with 4nM Calcein in PBS (Sigma-Aldrich, excitation wavelength 495nm and emission wavelength 515nm) at 37°C for 20 minutes. Cell

migration was quantified from digital images of the wells using the ImageJ software. The threshold of the image was adjusted and converted to a binary image. Using ImageJ a 2mm circle area was created and positioned in the empty central circle created by the stopper. The total area covered by the cells was quantified to determine the number of cells migrating. Experiments were done at least in triplicate.

37. Cell death was measured by quantifying DNA fragmentation using propidium iodide (Sigma-Aldrich) staining and FACS analysis as described before [78]. Briefly, both floating cells and attached cells were collected and fixed in 70% ethanol for one hour at -20^{0}C. The cells were centrifuged, washed with PBS, and incubated with propidium iodide (1µg/ml) and RNase (100µg/ml) for 30 minutes at room temperature.

38. Luciferase assays. The transcription factor response element activity was assessed in HCT116 (mtKRAS^{Hi}) and HKE3 (mtKRAS^{Lo}) cells co-transfected with luciferase constructs bearing response elements for STAT1 (4xGAS response element; Stratagene, #219091-51), STAT1/STAT2 (IRSE/interferon alpha response element; [79]) and STAT3 (4xm67 response element; [80]). All transfections were performed using polyethylenimine (Sigma, #48727). A CMV-β-gal plasmid was co-transfected as a control of transfection efficiency. 48 hours post transfection cells were stimulated with 10nm human EGF (Roche; #11376454001) for five hours before luciferase and β-gal activity were measured. Luciferase assays (Promega, #E4030) and β-galactosidase assays (Promega, #E2000) were performed according to the manufacturer's instructions using a SpectraMax M3 plate reader (Molecular Devices). Luciferase activity in arbitrary units was normalized againstβ-gal activity to correct for the transfection efficiency.

39. Transcriptional profiling of HCT116 (mtKRAS^{Hi}) and HKE3 (mtKRAS^{Lo}) cells with and without transforming growth factor alpha (TGFα) stimulation. For the stimulation studies, cells were seeded into 6 well plates until confluent. Cells were then starved for 18 hours and subsequently stimulated with TGF-α (0.01µg/ml, Abcam). RNA was isolated at 0, 15, 30, 60, 90 and 120 minutes post-stimulation using the TRIzol reagent (Thermo Fisher Scientific) according to the manufacturer's instructions. RNA was extracted from 3 biological replicates at each timepoint. RNA concentration was determined using the NanoDrop 2000 spectrophotometer (Thermo Fisher Scientific) and by Qubit assay (Thermo Fisher Scientific). RNA integrity was assessed using a Bioanalyzer 2100 (Agilent). Total RNA was converted to strand-specific Illumina compatible sequencing libraries using the NEXTflex Rapid Directional mRNA-Seq library Kit from BIOO Scientific (Austin, Texas) as per the manufacturer's instructions (v14.10). The cDNA libraries were PCR amplified for 15 cycles prior to assessment using a TapeStation 2200 (Agilent) for quality and Qubit fluorescence assay for quantification. In total, 36 cDNA libraries (2 cell lines x 6 time-points x 3 replicates per timepoint) were generated and sequenced on the Illumina HiSeq 2500 machine using a v2 High Output 100 cycle Kit (1x 100 bp SR). The quality and number of reads for each sample were assessed with FastQC v0.11.3. Adaptors were trimmed from reads, and low-quality bases with Phred scores < 28 were trimmed from ends of reads, using Trimgalore v0.4.0. Trimmed reads of less than 20 nucleotides were discarded. Reads passing all quality control steps were aligned to the hg38 assembly of the human genome using TopHat v2.1.0 [81], allowing for up to two mismatches. Reads not uniquely aligned to the genome were discarded. HTSeq-count v0.6.0 [82] was used in the union model to assign uniquely aligned reads to Ensembl Hg38.86-annotated genes. Data were normalized across libraries by the trimmed mean of M-values (TMM) normalization method, implemented in the R v3.2.1., Bioconductor package, EdgeR v3.10.2

[83]. Only genes that had at least 5 reads per million in at least one cell-line were further analyzed for evidence of differential gene expression. Differentially expressed genes were identified using the glm model implemented in EdgeR (FDR < 0.05). The RNAseq data were deposited in the Gene Expression Omnibus (GEO) under accession number GSE105094.

Supplementary References

1. Soh, J. *et al.* Oncogene mutations, copy number gains and mutant allele specific imbalance (MASI) frequently occur together in tumor cells. *PloS one* **4**, e7464 (2009).
2. Shirasawa, S., Furuse, M., Yokoyama, N. & Sasazuki, T. Altered growth of human colon cancer cell lines disrupted at activated Ki-ras. *Science (New York, N.Y.)* **260**, 85-88 (1993).
3. Kotlyar, M., Pastrello, C., Malik, Z. & Jurisica, I. IID 2018 update: context-specific physical protein-protein interactions in human, model organisms and domesticated species. *Nucleic Acids Res* **47**, D581-d589 (2019).
4. Kotlyar, M., Pastrello, C., Sheahan, N. & Jurisica, I. Integrated interactions database: tissue-specific view of the human and model organism interactomes. *Nucleic Acids Res* **44**, D536-541 (2016).
5. Brown, K. R. *et al.* NAViGaTOR: Network Analysis, Visualization and Graphing Toronto. *Bioinformatics* **25**, 3327-3329 (2009).
6. Fasterius, E. *et al.* A novel RNA sequencing data analysis method for cell line authentication. *PLoS One* **12**, e0171435 (2017).
7. Li, H. & Durbin, R. Fast and accurate short read alignment with Burrows-Wheeler transform. *Bioinformatics* **25**, 1754-1760 (2009).
8. Van der Auwera, G. A. *et al.* From FastQ data to high confidence variant calls: the Genome Analysis Toolkit best practices pipeline. *Current protocols in bioinformatics* **43**, 11.10.11-33 (2013).
9. McLaren, W. *et al.* The Ensembl Variant Effect Predictor. *Genome Biol* **17**, 122 (2016).
10. Chen, X. *et al.* Manta: rapid detection of structural variants and indels for germline and cancer sequencing applications. *Bioinformatics* **32**, 1220-1222 (2016).
11. Talevich, E., Shain, A. H., Botton, T. & Bastian, B. C. CNVkit: Genome-Wide Copy Number Detection and Visualization from Targeted DNA Sequencing. *PLoS computational biology* **12**, e1004873 (2016).
12. Li, H. Toward better understanding of artifacts in variant calling from high-coverage samples. *Bioinformatics* **30**, 2843-2851 (2014).
13. Wang, K., Li, M. & Hakonarson, H. ANNOVAR: functional annotation of genetic variants from high-throughput sequencing data. *Nucleic Acids Res* **38**, e164 (2010).
14. Kiel, C., Verschueren, E., Yang, J. S. & Serrano, L. Integration of protein abundance and structure data reveals competition in the ErbB signaling network. *Sci Signal* **6**, ra109 (2013).
15. Gloeckner, C. J., Boldt, K., Schumacher, A., Roepman, R. & Ueffing, M. A novel tandem affinity purification strategy for the efficient isolation and characterisation of native protein complexes. *Proteomics* **7**, 4228-4234 (2007).
16. Longo, P. A., Kavran, J. M., Kim, M. S. & Leahy, D. J. Transient mammalian cell transfection with polyethylenimine (PEI). *Methods in enzymology* **529**, 227-240 (2013).
17. Hilger, M. & Mann, M. Triple SILAC to determine stimulus specific interactions in the Wnt pathway. *Journal of proteome research* **11**, 982-994 (2012).
18. Chen, X., Wei, S., Ji, Y., Guo, X. & Yang, F. Quantitative proteomics using SILAC: Principles, applications, and developments. *Proteomics* **15**, 3175-3192 (2015).

[19] Chen, X. *et al.* Comparative Proteomic Study of Fatty Acid-treated Myoblasts Reveals Role of Cox-2 in Palmitate-induced Insulin Resistance. *Scientific reports* **6**, 21454 (2016).
[20] Park, S. S. *et al.* Effective correction of experimental errors in quantitative proteomics using stable isotope labeling by amino acids in cell culture (SILAC). *Journal of proteomics* **75**, 3720-3732 (2012).
[21] Turriziani, B. *et al.* On-beads digestion in conjunction with data-dependent mass spectrometry: a shortcut to quantitative and dynamic interaction proteomics. *Biology* **3**, 320-332 (2014).
[22] Boldt, K. *et al.* An organelle-specific protein landscape identifies novel diseases and molecular mechanisms. *Nature communications* **7**, 11491 (2016).
[23] Rappsilber, J., Ishihama, Y. & Mann, M. Stop and go extraction tips for matrix-assisted laser desorption/ionization, nanoelectrospray, and LC/MS sample pretreatment in proteomics. *Anal Chem* **75**, 663-670 (2003).
[24] Olsen, J. V. *et al.* Parts per million mass accuracy on an Orbitrap mass spectrometer via lock mass injection into a C-trap. *Mol Cell Proteomics* **4**, 2010-2021 (2005).
[25] Cox, J. & Mann, M. MaxQuant enables high peptide identification rates, individualized p.p.b.-range mass accuracies and proteome-wide protein quantification. *Nature biotechnology* **26**, 1367-1372 (2008).
[26] Teo, G. *et al.* SAINTexpress: improvements and additional features in Significance Analysis of INTeractome software. *Journal of proteomics* **100**, 37-43 (2014).
[27] Sowa, M. E., Bennett, E. J., Gygi, S. P. & Harper, J. W. Defining the human deubiquitinating enzyme interaction landscape. *Cell* **138**, 389-403 (2009).
[28] Bryan, K. *et al.* HiQuant: Rapid Postquantification Analysis of Large-Scale MS-Generated Proteomics Data. *Journal of proteome research* **15**, 2072-2079 (2016).
[29] Gloeckner, C. J., Boldt, K. & Ueffing, M. Strep/FLAG tandem affinity purification (SF-TAP) to study protein interactions. *Curr Protoc Protein Sci* **Chapter 19**, Unit19.20 (2009).
[30] Tyanova, S. *et al.* The Perseus computational platform for comprehensive analysis of (prote)omics data. *Nat Methods* **13**, 731-740 (2016).
[31] Doncheva, N. T., Assenov, Y., Domingues, F. S. & Albrecht, M. Topological analysis and interactive visualization of biological networks and protein structures. *Nature protocols* **7**, 670-685 (2012).
[32] Shannon, P. *et al.* Cytoscape: a software environment for integrated models of biomolecular interaction networks. *Genome research* **13**, 2498-2504 (2003).
[33] Ruepp, A. *et al.* CORUM: the comprehensive resource of mammalian protein complexes--2009. *Nucleic Acids Res* **38**, D497-501 (2010).
[34] Huttlin, E. L. *et al.* Architecture of the human interactome defines protein communities and disease networks. *Nature* **545**, 505-509 (2017).
[35] Rolland, T. *et al.* A proteome-scale map of the human interactome network. *Cell* **159**, 1212-1226 (2014).
[36] Hein, M. Y. *et al.* A human interactome in three quantitative dimensions organized by stoichiometries and abundances. *Cell* **163**, 712-723 (2015).
[37] Gupta, G. D. *et al.* A Dynamic Protein Interaction Landscape of the Human Centrosome-Cilium Interface. *Cell* **163**, 1484-1499 (2015).
[38] Coyaud, E. *et al.* Global Interactomics Uncovers Extensive Organellar Targeting by Zika Virus. *Mol Cell Proteomics* **17**, 2242-2255 (2018).
[39] Caron, E. *et al.* Precise Temporal Profiling of Signaling Complexes in Primary Cells Using SWATH Mass Spectrometry. *Cell reports* **18**, 3219-3226 (2017).
[40] Nakhaeizadeh, H., Amin, E., Nakhaei-Rad, S., Dvorsky, R. & Ahmadian, M. R. The RAS-Effector Interface: Isoform-Specific Differences in the Effector Binding Regions.

PloS one **11**, e0167145 (2016).
41 Linnemann, T., Zheng, Y.-H., Mandic, R. & Matija Peterlin, B. Interaction between Nef and Phosphatidylinositol-3-Kinase Leads to Activation of p21-Activated Kinase and Increased Production of HIV. *Virology* **294**, 246-255 (2002).
42 Harjes, E. *et al.* GTP-Ras Disrupts the Intramolecular Complex of C1 and RA Domains of Nore1. *Structure* **14**, 881-888 (2006).
43 Rudolph, M. G. *et al.* Thermodynamics of Ras/Effector and Cdc42/Effector Interactions Probed by Isothermal Titration Calorimetry. *Journal of Biological Chemistry* **276**, 23914-23921 (2001).
44 Wang, J., Peng, X., Li, M. & Pan, Y. Construction and application of dynamic protein interaction network based on time course gene expression data. *Proteomics* **13**, 301-312 (2013).
45 Wohlgemuth, S. *et al.* Recognizing and Defining True Ras Binding Domains I: Biochemical Analysis. *Journal of Molecular Biology* **348**, 741-758 (2005).
46 Azeloglu, E. U. & Iyengar, R. Good practices for building dynamical models in systems biology. *Sci Signal* **8**, fs8 (2015).
47 Kholodenko, B., Yaffe, M. B. & Kolch, W. Computational approaches for analyzing information flow in biological networks. *Sci Signal* **5**, re1 (2012).
48 Sobie, E. A., Lee, Y. S., Jenkins, S. L. & Iyengar, R. Systems biology--biomedical modeling. *Sci Signal* **4**, tr2 (2011).
49 Morris, M. K., Saez-Rodriguez, J., Sorger, P. K. & Lauffenburger, D. A. Logic-based models for the analysis of cell signaling networks. *Biochemistry* **49**, 3216-3224 (2010).
50 Stojmirovic, A. & Yu, Y. K. Information flow in interaction networks. *J Comput Biol* **14**, 1115-1143 (2007).
51 Stojmirovic, A. & Yu, Y. K. Information flow in interaction networks II: channels, path lengths, and potentials. *J Comput Biol* **19**, 379-403 (2012).
52 Breuer, K. *et al.* InnateDB: systems biology of innate immunity and beyond--recent updates and continuing curation. *Nucleic Acids Res* **41**, D1228-1233 (2013).
53 Stojmirovic, A., Bliskovsky, A. & Yu, Y. K. CytoITMprobe: a network information flow plugin for Cytoscape. *BMC Res Notes* **5**, 237 (2012).
54 Heinz, S. *et al.* Simple combinations of lineage-determining transcription factors prime cis-regulatory elements required for macrophage and B cell identities. *Mol Cell* **38**, 576-589 (2010).
55 Orchard, S. *et al.* Protein interaction data curation: the International Molecular Exchange (IMEx) consortium. *Nat Methods* **9**, 345-350 (2012).
56 Kanehisa, M. & Goto, S. KEGG: kyoto encyclopedia of genes and genomes. *Nucleic Acids Res* **28**, 27-30 (2000).
57 Croft, D. *et al.* The Reactome pathway knowledgebase. *Nucleic Acids Res* **42**, D472-477 (2014).
58 Gene Ontology, C. Gene Ontology Consortium: going forward. *Nucleic Acids Res* **43**, D1049-1056 (2015).
59 Forbes, S. A. *et al.* COSMIC: somatic cancer genetics at high-resolution. *Nucleic Acids Res* **45**, D777-D783 (2017).
60 Rose, P. W. *et al.* The RCSB protein data bank: integrative view of protein, gene and 3D structural information. *Nucleic Acids Res* **45**, D271-D281 (2017).
61 Law, V. *et al.* DrugBank 4.0: shedding new light on drug metabolism. *Nucleic Acids Res* **42**, D1091-1097 (2014).
62 Yates, A. *et al.* Ensembl 2016. *Nucleic Acids Res* **44**, D710-716 (2016).
63 Gholami, A. M. *et al.* Global proteome analysis of the NCI-60 cell line panel. *Cell reports* **4**, 609-620 (2013).

64 Geiger, T., Wehner, A., Schaab, C., Cox, J. & Mann, M. Comparative proteomic analysis of eleven common cell lines reveals ubiquitous but varying expression of most proteins. *Mol Cell Proteomics* **11**, M111 014050 (2012).
65 Frias, S., Bryan, K., Brinkman, F. S. & Lynn, D. J. CerebralWeb: a Cytoscape.js plug-in to visualize networks stratified by subcellular localization. *Database (Oxford)* **2015**, bav041 (2015).
66 Goenawan, I. H., Bryan, K. & Lynn, D. J. DyNet: visualization and analysis of dynamic molecular interaction networks. *Bioinformatics* **32**, 2713-2715 (2016).
67 Zhang, B. *et al.* Comprehensive molecular characterization of human colon and rectal cancer. *Nature* **487**, 330-337 (2012).
68 Singh, R. & Mukhopadhyay, K. Survival analysis in clinical trials: Basics and must know areas. *Perspectives in clinical research* **2**, 145-148 (2011).
69 Tibshirani, R. Regression Shrinkage and Selection via the Lasso. *Journal of the Royal Statistical Society. Series B (Methodological)* **58**, 267-288 (1996).
70 Robin, X. *et al.* pROC: an open-source package for R and S+ to analyze and compare ROC curves. *BMC bioinformatics* **12**, 77 (2011).
71 Roskoski, R., Jr. The ErbB/HER family of protein-tyrosine kinases and cancer. *Pharmacological research* **79**, 34-74 (2014).
72 Lu, B., McClatchy, D. B., Kim, J. Y. & Yates, J. R., 3rd. Strategies for shotgun identification of integral membrane proteins by tandem mass spectrometry. *Proteomics* **8**, 3947-3955 (2008).
73 Sokolina, K. *et al.* Systematic protein-protein interaction mapping for clinically relevant human GPCRs. *Molecular systems biology* **13**, 918 (2017).
74 Yao, Z. *et al.* A Global Analysis of the Receptor Tyrosine Kinase-Protein Phosphatase Interactome. *Mol Cell* **65**, 347-360 (2017).
75 Deribe, Y. L. *et al.* Regulation of epidermal growth factor receptor trafficking by lysine deacetylase HDAC6. *Sci Signal* **2**, ra84 (2009).
76 Guzman, C., Bagga, M., Kaur, A., Westermarck, J. & Abankwa, D. ColonyArea: an ImageJ plugin to automatically quantify colony formation in clonogenic assays. *PloS one* **9**, e92444 (2014).
77 Dhillon, A. S. *et al.* A Raf-1 mutant that dissociates MEK/extracellular signal-regulated kinase activation from malignant transformation and differentiation but not proliferation. *Mol Cell Biol* **23**, 1983-1993 (2003).
78 O'Neill, E., Rushworth, L., Baccarini, M. & Kolch, W. Role of the kinase MST2 in suppression of apoptosis by the proto-oncogene product Raf-1. *Science* **306**, 2267-2270 (2004).
79 John, S., Vinkemeier, U., Soldaini, E., Darnell, J. E., Jr. & Leonard, W. J. The significance of tetramerization in promoter recruitment by Stat5. *Mol Cell Biol* **19**, 1910-1918 (1999).
80 Horvath, C. M., Wen, Z. & Darnell, J. E., Jr. A STAT protein domain that determines DNA sequence recognition suggests a novel DNA-binding domain. *Genes & development* **9**, 984-994 (1995).
81 Kim, D. *et al.* TopHat2: accurate alignment of transcriptomes in the presence of insertions, deletions and gene fusions. *Genome Biol* **14**, R36 (2013).
82 Anders, S., Pyl, P. T. & Huber, W. HTSeq--a Python framework to work with high-throughput sequencing data. *Bioinformatics* **31**, 166-169 (2015).
83 Robinson, M. D., McCarthy, D. J. & Smyth, G. K. edgeR: a Bioconductor package for differential expression analysis of digital gene expression data. *Bioinformatics* **26**, 139-140 (2010).
