# Supplementary material for: Extensive rewiring of the EGFR network in colorectal cancer cells expressing transforming levels of KRASG13D
Source: Nat Commun. 2020 Jan 24;11:499. doi: 10.1038/s41467-019-14224-9 (PMC6981206; doi:10.1038/s41467-019-14224-9)
Supplement: Supplementary file 3 — Reporting Summary [file 41467_2019_14224_MOESM3_ESM.pdf]

nature research

Corresponding author(s): Walter Kolch, David Lynn, Karsten Boldt

Last updated by author(s): Sep 4, 2019

Reporting Summary

Nature Research wishes to improve the reproducibility of the work that we publish. This form provides structure for consistency and transparency in reporting. For further information on Nature Research policies, see Authors & Referees and the Editorial Policy Checklist .

Statistics

For all statistical analyses, confirm that the following items are present in the figure legend, table legend, main text, or Methods section.

n/a	Confirmed	
☐	☒	The exact sample size (*n*) for each experimental group/condition, given as a discrete number and unit of measurement
☐	☒	A statement on whether measurements were taken from distinct samples or whether the same sample was measured repeatedly
☐	☒	The statistical test(s) used AND whether they are one- or two-sided<br>*Only common tests should be described solely by name; describe more complex techniques in the Methods section.*
☐	☒	A description of all covariates tested
☐	☒	A description of any assumptions or corrections, such as tests of normality and adjustment for multiple comparisons
☐	☒	A full description of the statistical parameters including central tendency (e.g. means) or other basic estimates (e.g. regression coefficient) AND variation (e.g. standard deviation) or associated estimates of uncertainty (e.g. confidence intervals)
☐	☒	For null hypothesis testing, the test statistic (e.g. *F*, *t*, *r*) with confidence intervals, effect sizes, degrees of freedom and *P* value noted<br>*Give P values as exact values whenever suitable.*
☒	☐	For Bayesian analysis, information on the choice of priors and Markov chain Monte Carlo settings
☐	☒	For hierarchical and complex designs, identification of the appropriate level for tests and full reporting of outcomes
☒	☐	Estimates of effect sizes (e.g. Cohen's *d*, Pearson's *r*), indicating how they were calculated

Our web collection on statistics for biologists contains articles on many of the points above.

Software and code

Policy information about availability of computer code

Data collection	Analysis of AP-MS data: The resulting mass spectra were analyzed using the MaxQuant software suite 18 (v 1.3.0.5) containing the in-built Andromeda search engine to identify the proteins from the UniProt HUMAN database (release 2014_02) containing 20,242 entries. The following MaxQuant parameters were used: trypsin was selected as enzyme, with two missed cleavages per peptide allowed; fixed amino acids modification was carboamidomethylation of cysteines; variable amino acids modifications were oxidation in methionine and acetylation in protein N-terminus; first search 20ppm and main search 6ppm with fragment ion mass tolerance set to 0.5 Da; 0.01 False Discovery Rate for analyses at both peptide and protein levels.
Data analysis	MS data were analysed using Maxquant, MASCOT, HiQuant and SAINTexpress. Matlab was used to generate the RAS binder model. The Genome Analysis Toolkit pipelines (GATK, v4.1.30) were used for whole genome sequence analysis. Impact of genetic variants was predicted with the Ensembl (release 97) Variant Effect Predictor. Gene Ontology (GO) and pathway analyses were performed using InnateDB.com. Transcription factor binding site analysis was undertaken using the findMotifs.pl program in HOMER v4.8. Kaplan-Meier curves were plotted using PRISM 7.0.3. All software and their uses are referenced in the manuscript methods. We used Mathematica to generate Figure 5A; Cytoscape for Supplementary Figures 2A, 5C-F, 6A, 6B, and 9B; and R for Supplementary Figures 3A and 9B.

For manuscripts utilizing custom algorithms or software that are central to the research but not yet described in published literature, software must be made available to editors/reviewers. We strongly encourage code deposition in a community repository (e.g. GitHub). See the Nature Research guidelines for submitting code & software for further information.

Data

Policy information about availability of data

All manuscripts must include a data availability statement. This statement should provide the following information, where applicable:

- Accession codes, unique identifiers, or web links for publicly available datasets
- A list of figures that have associated raw data
- A description of any restrictions on data availability

All AP-MS PPI data generated in this study have been deposited to the International Molecular Exchange (IMEx) Database (IMEx accession number IM-26434 -

private pending publication). PPI data can also be explored at primesdb.eu. Genome sequencing data have been submitted to the NCBI sequence read archive under accession number PRJNA374513. RNAseq data were deposited in the Gene Expression Omnibus (GEO) under accession number GSE105094.
List of figures with associated raw data: Figures 2-6, S1-S5, S8.
There are no restrictions on data availability.

Field-specific reporting

Please select the one below that is the best fit for your research. If you are not sure, read the appropriate sections before making your selection.

[x] Life sciences [ ] Behavioural & social sciences [ ] Ecological, evolutionary & environmental sciences

For a reference copy of the document with all sections, see nature.com/documents/nr-reporting-summary-flat.pdf

Life sciences study design

All studies must disclose on these points even when the disclosure is negative.

Sample size	Network reconstruction: We analyzed 95 bait and empty vector control immunoprecipitates (IPs) in 2 different cell lines from both forward and reverse SILAC labelled cells using three biological and two technical replicates per bait resulting in 1,710 samples and 1,140 qMS analyses.
Data exclusions	To perform protein quantification the following criteria needed to be met: a minimum of one unique peptide with a minimum length of six amino acids, and a minimum ratio count of two. Proteins not meeting these criteria were excluded. Contaminant proteins were also excluded by enabling the MaxQuant contaminant database search.
Replication	All experiments were successfully replicated at least 3 times.
Randomization	Our experimental design reconstructed the EGFR network in 2 different cell lines, it was therefore not possible to randomize samples to different groups.
Blinding	The investigators were not blinded to the group allocations.

Reporting for specific materials, systems and methods

We require information from authors about some types of materials, experimental systems and methods used in many studies. Here, indicate whether each material, system or method listed is relevant to your study. If you are not sure if a list item applies to your research, read the appropriate section before selecting a response.

Materials & experimental systems

n/a	Involved in the study	
[ ]	[x]	Antibodies
[ ]	[x]	Eukaryotic cell lines
[x]	[ ]	Palaeontology
[x]	[ ]	Animals and other organisms
[x]	[ ]	Human research participants
[x]	[ ]	Clinical data

Methods

n/a	Involved in the study	
[x]	[ ]	ChIP-seq
[x]	[ ]	Flow cytometry
[x]	[ ]	MRI-based neuroimaging

Antibodies

Antibodies used	K-RAS (sc-30), Tub TU-02 (sc-8035), PTK6 C18 (sc-1188), PRPS1/2/L (sc-292588), CAV2 (sc-7942), CDC42 (sc-87), RSK1/ RPS6KA1 (sc-231), RKIP/PEBP1 (sc-28837), PRKCA (sc-20), PRKCZ (sc-17781), BAG2 (sc-366091), and CAV1 (sc-894) from Santa Cruz; GAPDH 1410C (2118S), Paxillin (2542S), Vinculin (4650), BAD (9292), phospho-BAD S112 (9296), phospho-AKT (9271S), AKT (9272S), NCK1 (2319), SRC (2123), MAPK7/ERK5 (3372) from Cell Signaling. ERK1/2 (M5670), phospho-ERK1/2 (M8159), anti-Flag M2 peroxidase (A8952), protein G-sepharose, anti-FLAG-M2 conjugated agarose beads (A2220), DUSP4 (SAB1403748) and Calcein (C1359) were from Sigma-Aldrich. STK38/NDR1 (BD 610828) and IQGAP1 (BD 610611) were from BD Biosciences. RAC1 (05-389) was from EMD Millipore. CSNK1/2 (ab10474) and CDC37 (ab56598) were from Abcam.
Validation	Antibody validation was provided by vendors, and further validated as fit for purpose by immunoprecipitation and Western blotting as described in the manuscript and Fig. S3B.

Eukaryotic cell lines

Policy information about cell lines

Cell line source(s)	HCT116 and HKE3 cells were obtained from Dr Shirasawa and are described in the following publication: Shirasawa, S., Furuse, M., Yokoyama, N. & Sasazuki, T. Altered growth of human colon cancer cell lines disrupted at activated Ki-ras. Science 260, 85-88 (1993)
Authentication	Cell lines were authenticated by whole genome sequencing (as reported in this manuscript) and RNAseq (Fasterius, E. et al. A novel RNA sequencing data analysis method for cell line authentication. PloS one 12, e0171435 (2017)).
Mycoplasma contamination	Cell lines were routinely (every 3 months) tested for mycoplasma contamination with negative results.
Commonly misidentified lines (See ICLAC register)	n/a
