# Supplementary material for: Extensive rewiring of the EGFR network in colorectal cancer cells expressing transforming levels of KRASG13D
Source: Nat Commun. 2020 Jan 24;11:499. doi: 10.1038/s41467-019-14224-9 (PMC6981206; doi:10.1038/s41467-019-14224-9)
Supplement: Supplementary file 4 — Description of Additional Supplementary Files [file 41467_2019_14224_MOESM4_ESM.pdf]

Description of Additional Supplementary Files

File Name: Supplementary Data 1
Description: **95 bait proteins analyzed in the mtKRAS^{Hi} and mtKRAS^{Lo} cell lines.**

File Name: Supplementary Data 2
Description: **Bait-prey interactions detected in the EGFRNet$^{mtKRAS-Hi}$ and EGFRNet$^{mtKRAS-Lo}$ protein-protein interaction networks (PPINs).** Interactions colored in red or green represent interactions present in EGFRNet$^{mtKRAS-Hi}$ or EGFRNet$^{mtKRAS-Lo}$ that were significantly rewired.

File Name: Supplementary Data 3
Description: **The ERBB1-4 MYTH interactome.**

File Name: Supplementary Data 4
Description: **A union network of all bait-prey interactions detected in EGFRNet$^{mtKRAS-Hi}$ and/or EGFRNet$^{mtKRAS-Lo}$.** Rows colored in red are interactions enhanced in mtKRAS^{Hi}. Rows colored in green correspond to enhanced interactions in mtKRAS^{Lo}.

File Name: Supplementary Data 5
Description: **Rewired interactions**. Bait-prey interactions where the prey abundance is significantly different in the mtKRAS^{Hi} bait immunoprecipitates (IPs) compared to the corresponding IPs from mtKRAS^{Lo} cells (P value ≤ 0.05 and Significance A ≤ 0.05).

File Name: Supplementary Data 6
Description: **Genes in impact SNVs.** Single nucleotide variants (SNVs) variant impact analysis of HCT116 and HKE3 cell using GATK joint variant calling (Van der Auwera et al. Current protocols in bioinformatics 43, 11.10.11-33, 2013). All variants with genotype depths >10x were extracted and variant impact assessed using Ensembl Variant Effect Predictor (McLaren, W. et al. Genome Biol 17: 122, 2016). Discordant high and medium impact variants were extracted and genes containing variants identified. Discordant genes containing SNVs were then filtered to find genes that were expressed on average >1 count per million across both cell lines, located network nodes and rewired.

File Name: Supplementary Data 7
Description: **Genes in Structural Variants.** Structural variant (SV) identified between HCT116 and HKE3 cell-lines using Manta (Chen et al. Bioinformatics 32, 1220, 2016). Genes found within discordant SVs were then filtered to find genes that were expressed on average >1 count per million across both cell lines, differentially expressed and abundant, located network nodes and rewired.

File Name: Supplementary Data 8
Description: **Genes in CNVs.** Copy number Variation (CNV) analysis of HCT116 and HKE3 cell-lines using CNVkit (Talevich et al. PLoS Computational Biology 12, e1004873, 2016). CNVs segments were called using HCT116 as the normal sample, and then discordant genes within CNVs were identified in CNVs with a greater than -/+0.4 log2 coverage value. Discordant genes were then filtered to find genes that were expressed on average >1 count per million across both cell lines, located network nodes and rewired.

File Name: Supplementary Data 9

Description: **Differentially expressed (DE) proteins in mtKRAS^{Hi} vs mtKRAS^{Lo} cells.** The results of pathway analysis of differentially expressed proteins are also shown.

File Name: Supplementary Data 10
Description: **Differentially phosphorylated (DP) proteins in mtKRAS^{Hi} vs mtKRAS^{Lo} cells.** The results of pathway analysis of differentially phosphorylated proteins are also shown.

File Name: Supplementary Data 11
Description: **Pathway analysis of rewired nodes using InnateDB.com.**

File Name: Supplementary Data 12
Description: **CORUM complexes identified in the EGFRNet$^{mtKRAS\text{-}Hi}$ and EGFRNet$^{mtKRAS\text{-}Lo}$ protein-protein interaction networks (PPINs).** Complexes where at least 70% of the complex component proteins are nodes in one or both of the networks are shown.

File Name: Supplementary Data 13
Description: **RAS effector ranking.**

File Name: Supplementary Data 14
Description: **Information flow scores for nodes in EGFRNet$^{mtKRAS\text{-}Hi}$ and EGFRNet$^{mtKRAS\text{-}Lo}$**

File Name: Supplementary Data 15
Description: **The top rewired baits are prognostic for CRC survival.** The top and bottom rewired baits were identified based on the number of rewired interactions identified per bait. Where two baits had the same number of rewired interactions they were ranked based on the sum of the SILAC ratios for all of their rewired preys. Alterations in the top 20 and bottom 20 rewired bait proteins identified in the TCGA Colorectal Adenocarcinoma dataset of 629 patients are also shown in this table, as is the corresponding survival data for patients with/without alterations in the top 20 or bottom 20 rewired bait proteins.

File Name: Supplementary Software 1
Description: Mathematica code for Figure 5A.

File Name: Supplementary Software 2
Description: Cytoscape session file for Supplementary Figure 2A.

File Name: Supplementary Software 3
Description: R-code and source data used for Supplementary Figure 3A.

File Name: Supplementary Software 4
Description: Cytoscape session file for Supplementary Figures 5C,D.

File Name: Supplementary Software 5
Description: Cytoscape session file for Supplementary Figures 5E,F.

File Name: Supplementary Software 6
Description: Cytoscape session file for Supplementary Figures 6A,B.

File Name: Supplementary Software 7
Description: R-script for Supplementary Figure 9B.
