# Supplementary material for: Extensive rewiring of the EGFR network in colorectal cancer cells expressing transforming levels of KRASG13D
Source: Nat Commun. 2020 Jan 24;11:499. doi: 10.1038/s41467-019-14224-9 (PMC6981206; doi:10.1038/s41467-019-14224-9)
Supplement: Supplementary file 25 — Supplementary Software 6 [file 41467_2019_14224_MOESM25_ESM.zip › Supplementary Figure 6AB_code/Supplementary Figure 6AB_Readme.docx]

This file contains the Cytoscape session file for Supplementary Figures 6A,B
